# Influence of the carbazole moiety in self-assembling molecules as selective contacts in perovskite solar cells: interfacial charge transfer kinetics and solar-to-energy efficiency effects†

**DOI:** 10.1039/d3na00811h

**Published:** 2023-10-16

**Authors:** Dora A. González, Carlos E. Puerto Galvis, Wenhui Li, Maria Méndez, Ece Aktas, Eugenia Martínez-Ferrero, Emilio Palomares

**Affiliations:** a Institute of Chemical Research of Catalonia (ICIQ-CERCA) Avda. Països Catalans 16 43007 Tarragona Spain epalomares@iciq.es; b Department of Electric, Electronic and Automatic Engineering, Universitat Rovira i Virgili Avda. Països Catalans 26 43007 Tarragona Spain; c ICREA Passeig Lluís Companys 23 08010 Barcelona Spain

## Abstract

The use of self-assembled molecules (SAMs) as hole transport materials (HTMs) in p–i–n perovskite solar cells (iPSCs) has triggered widespread research due to their relatively easy synthetic methods, suitable energy level alignment with the perovskite material and the suppression of chemical defects. Herein, three new SAMs have been designed and synthesised based on a carbazole core moiety and modified functional groups through an efficient synthetic protocol. The SAMs have been used to understand the SAM/perovskite interface interactions and establish the relationship between the SAM molecular structure and the resulting performance of the perovskite-based devices. The best devices show efficiencies ranging from 18.9% to 17.5% under standard illumination conditions, which are very close to that of our benchmark EADR03, which has been recently commercialised. Our work aims to provide knowledge on the structure of the molecules *versus* device function relationship.

## Abstract 2

The linker and the terminal functional groups of SAMs based on the carbazole core play an important role in the efficiency of inverted perovskite solar cells.

## Graphical abstract

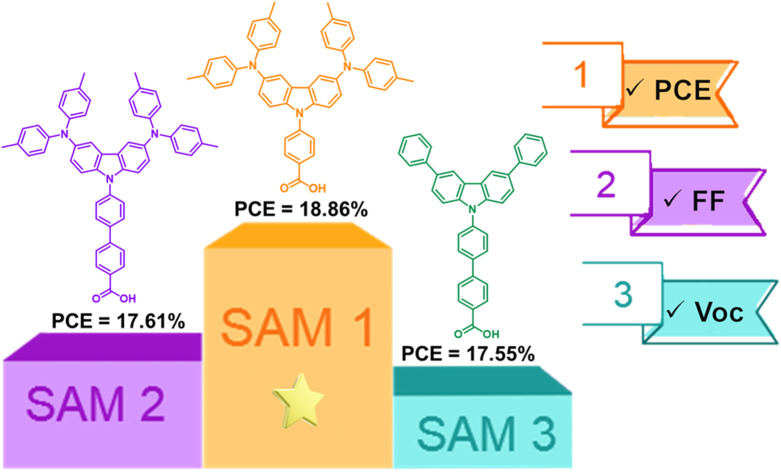

## Introduction

Over the last decade, the power conversion efficiency (PCE) of perovskite solar cells has increased from 3.8% to 25.7% for the standard configuration (n–i–p) and to 24.2% for the inverted design (p–i–n).^1,2^ This impressive progress has been achieved thanks to the research on tuning the perovskite semiconductor composition, improving the defect's passivation, and optimising the charge transporting layers, among other factors.^3^ Among them, the charge transporting materials, including the electron transporting material (ETM) and hole transporting material (HTM), play a key role in achieving such efficiencies.^4^ The synthesis and design of HTMs have become an important topic among the perovskite research community. Recently, a new family of HTMs known as self-assembled molecules (SAMs) has attracted tremendous attention as hole selective contacts in inverted perovskite solar cells (PSCs).^5^ SAMs present several advantages in comparison to the widely used poly[bis(4-phenyl) (2,4,6-trimethylphenyl) amine] (PTAA) polymer, for instance, in terms of costs, reproducibility, and stability.^5–7^ Furthermore, SAM-based iPSCs have achieved over 24% efficiency,^8^ confirming that SAMs are optimal hole-selective contacts in high-performance devices.

SAMs are composed of three different parts: (i) anchoring groups, in particular, the phosphonic acid and carboxylic acid groups, (ii) linker groups, made of alkyl chains or conjugated benzenes, and (iii) functional groups, based on amines, thiols, or carbazoles.^9,10^ The anchoring groups chemically bond to ITO to form a robust and uniform monolayer. The linker groups determine the charge transport properties, molecular packing, and geometry. The functional groups interact with the perovskite layer, improving the surface coverage and the passivation of defects, promoting the growth of the perovskite film, and inducing changes in the surface work function.^11,12^

Recently, it has been shown that the application of well-known electron-rich groups as functional groups in the SAM structure can positively impact the performance of the devices.^13^ In particular, a carbazole core is widely adapted for the synthesis of new materials for solar cells. The first use of a carbazole core in SAMs as a dopant-free hole selective contact (HSC) was implemented in 2018 by Magomedov *et al.*, reaching a PCE of more than 17%. Al-Ashouri *et al.*^14^ designed two new SAMs (MeO-2PACz and 2PACz) to create an energetically aligned interface for three different perovskite compositions, allowing the reduction of non-radiative recombination at the interface between the perovskite and the contact layer, reaching PCEs of up to 20%. The results highlight that carbazole derivatives can combine all the necessary features to reduce interfacial charge losses and are excellent candidates for further chemical engineering of high-performance hole-selective contacts. Yalcin *et al.*^15^ demonstrated the influence of the SAMs on the surface properties of indium tin oxide (ITO) and their beneficial role in improving the perovskite performance. Later, other studies undertaken on SAMs, reported by our group and other authors,^10,16–19^ consider that the PCE for most of these devices relies on the molecular structure of the SAM. Therefore, the search for a suitable SAM material to increase the PCE, including reliable passivation of defects, proper alignment of the energy levels, good operating stability, and fast charge transport, is still needed.

**Scheme 1:**
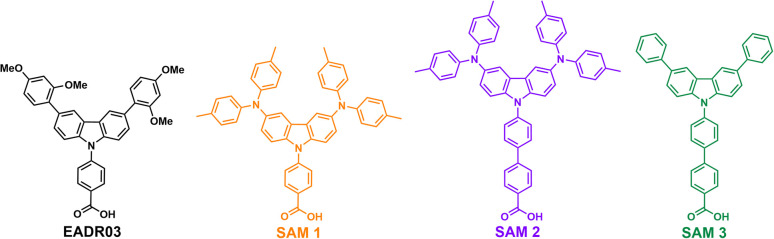
Molecular structure of EADR03 and the three novel SAM molecules used as hole selective contacts in iPSCs.

This work has designed and synthesised three new SAMs with carboxylic acid (–COOH) as the anchoring group, phenyl or biphenyl moieties as a linker, and different carbazole core-based functional groups. See Scheme 1 for the EADR03 – commercial molecule synthesised by our group and used as a reference in this work – and the three new molecules named SAM1, SAM2 and SAM3. We have prepared devices and analysed the interfacial processes between the three new carbazole-based molecules as a SAM and its adjacent layers (perovskite and ITO) to get a deeper insight into the effect of the different molecular structures on the PCE.

## Results and discussion

**Fig. 1:**
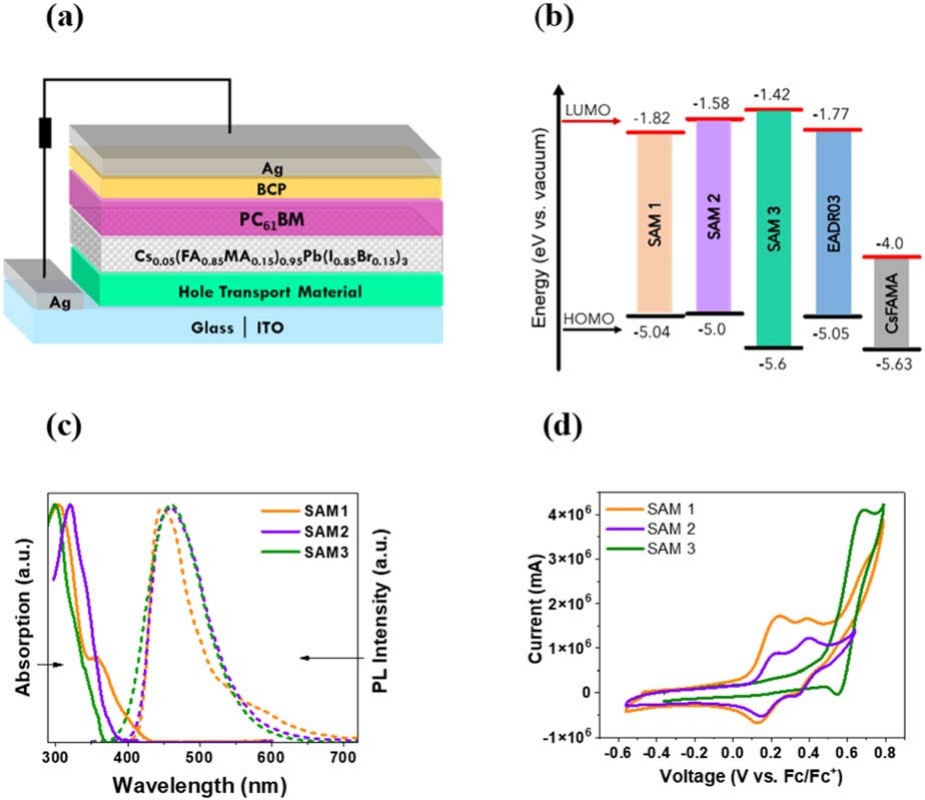
(a) The architecture of the p–i–n device used in this work, (b) the energy levels of the SAMs, EADR03 and perovskite, (c) UV-vis absorption and emission spectra of conjugated SAMs in DCM solution, and (d) cyclic voltammetry results of conjugated SAMs in the supporting electrolyte (TBAPF_6_ in DCM), measured using ferrocene (Fc/Fc^+^) as an internal reference.

SAMs were used as HTMs in iPSCs based on a triple cation perovskite (Cs_0.05_(FA_0.85_MA_0.15_)_0.95_Pb(I_0.85_Br_0.15_)_3_) labelled CsFAMA.^20^ The device structure of the iPSC presented in this study consists of ITO/SAM/CsFAMA/PC_61_BM/BCP/Ag, as shown in Fig. 1a.

The synthetic details of the three SAMs can be found in the ESI† and the measures to determine their structures using ^1^H and ^13^C NMR spectroscopy. The energy levels of the SAMs and CsFAMA are illustrated in Fig. 1b, where the energy levels for CsFAMA and EADR03 were obtained from the literature.^16^ The energy levels of SAM1, SAM2 and SAM3 were estimated using absorption and photoluminescence spectroscopy in combination with cyclic voltammetry (see results in Fig. 1c and d and ESI† for experimental details). The energetic alignment between SAMs and perovskite is a critical topic that has already been investigated from the molecular design perspective^12,21^

The UV-visible absorption and photoluminescence (PL) spectra were recorded for the three new molecules in solution (10^−5^ M in dichloromethane, DCM), see Fig. 1c. All the samples show a broad absorption band ranging from 290 to 400 nm, which is assigned to the π–π* transitions.^5^ The PL emission spectra show a wide emission band with the maximum at 452 nm for SAM1, 446 nm for SAM2 and 470 nm for SAM3. The optical gap of SAMs was obtained from the absorption edge wavelength using UV-vis measurements (see Table S1†).^22^

Cyclic voltammetry was performed to calculate the HOMO values (see Fig. 1d).^23^SAM1 and SAM2 exhibit two reversible oxidation waves assigned to the oxidation, mainly, from the di-*p*-tolylamine groups, while SAM3 shows only one wave due to the carbazole-core. The introduction of phenyl groups into SAM3 leads to a deeper HOMO value of −5.6 eV compared to −5.04 for SAM1 and −5.0 for SAM2. In accordance with the results, the energy levels of the new SAMs are suitable with respect to the CsFAMA energy level and very similar to those of EADR03.

The thermal behaviour of SAM1, SAM2 and SAM3 was analyzed by thermogravimetric analysis (TGA) (Fig. S1 and Table S1†). The thermogram indicates that the decomposition temperature (*T*_dec_) is higher than the annealing temperature applied during the fabrication of the devices, making all the SAMs suitable for their application in iPSCs.

**Fig. 2:**
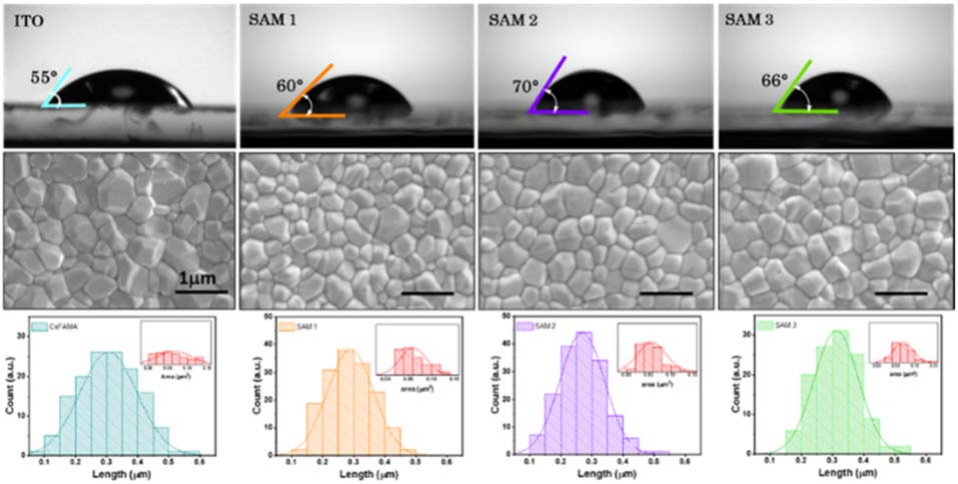
Contact angle measurements on the different SAM surfaces and ITO, and the FESEM top view of perovskite films deposited on (a) ITO, (b) ITO/SAM1, (c) ITO/SAM2 and (d) ITO/SAM3. All scale bars are 1 μm.

The surface wettability of the ITO-coated substrates was investigated by measuring the contact angle with water. This technique allows the detection of changes in the ITO surface because the deposition of an additional layer will affect the contact angle values of ITO.^9^ The contact angles on SAM1, SAM2 and SAM3 are estimated to be 60°, 70° and 66°, respectively (see Fig. 2 top images), which shows a higher hydrophobicity character in comparison to EADR03 estimated at 50°.^16^ Furthermore, the contact angle of bare ITO is estimated to be 55°, indicating that SAMs cover the ITO surface. To corroborate the homogeneous deposition of the perovskite solution on SAM layers, we employed field emission scanning electron microscopy (FESEM), as shown in Fig. 2 bottom images. We observed a similar perovskite grain size distribution for ITO and ITO/SAM3, whereas for ITO/SAM1 and ITO/SAM2 the grain size slightly decreases (see Table S2†).

**Fig. 3:**
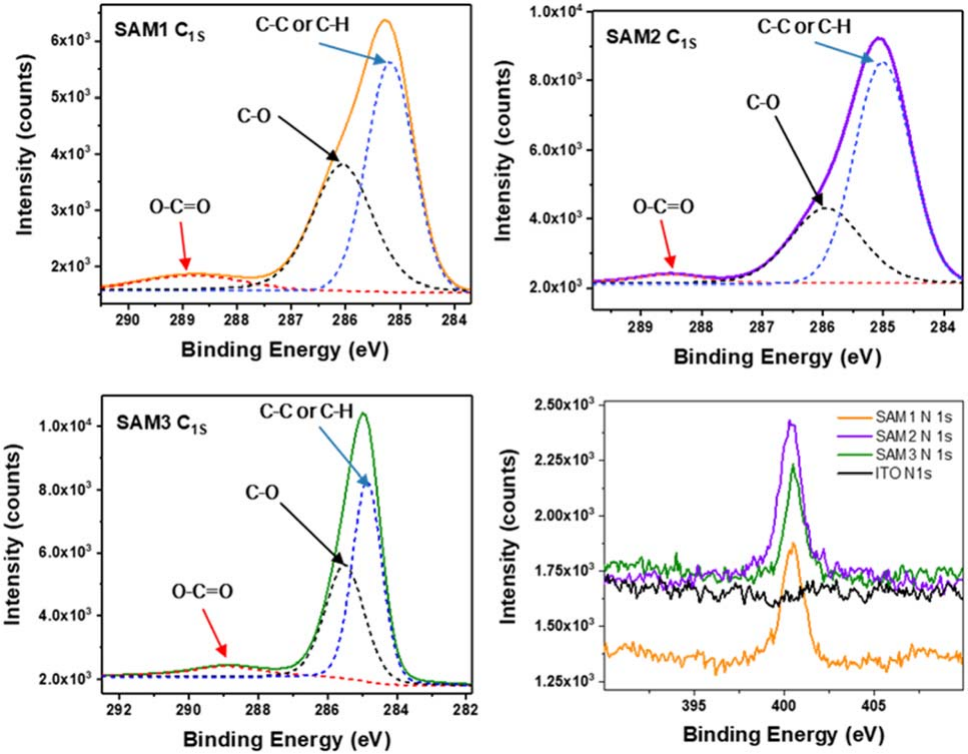
X-ray photoelectron spectroscopy of C1s and N1s for SAMs on the treated ITO.

To further corroborate the surface modification on the ITO by SAM molecules, X-ray photoelectron spectroscopy (XPS) was used to examine the atomic bonds of SAMs on the metal oxide surface (see Fig. 3, S2 and Table S3†). The C1s spectra were decomposed into 3 peaks assigned to C–C or C–H (285 eV), C–O (286 eV) and COOCH (289 eV) bonds. The N1s spectra show the same peak position at 400 eV for all the SAMs assigned to the C–N bond, which is not observed on the bare ITO. This confirms the successful formation of the self-assembled monolayer on the ITO.

**Fig. 4:**
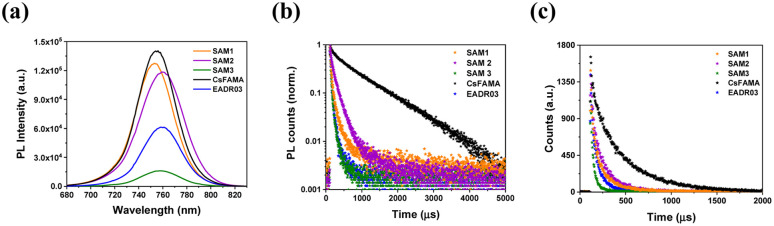
(a) Steady-state photoluminescence spectra and (b) normalized time-resolved photoluminescence decays with a fixed 5000 acquisition counts and (c) with a fixed time at 300 seconds. The samples were excited from the glass side (635 nm) at 770 nm.

We examined the charge transfer process at the perovskite/HTM interfaces performing steady-state (PL) and time-resolved photoluminescence analyses (TRPL), as shown in Fig. 4. The PL spectra show a peak in the region around 760 nm for perovskite, and as can be seen, when perovskite was deposited on a SAM layer, the emission shows a significant quenching compared to the bare perovskite film, Fig. 4a. This confirms the injection of holes from the valence band of the CsFAMA layer to the HOMO of the SAM. However, the PL spectrum of SAM1 and SAM2 based perovskite films exhibits a stronger PL emission than SAM3, suggesting that the addition of the phenyl moiety to SAM3 helps promote hole transfer more efficiently. The TRPL decay curves of Fig. 4b were fitted with a double-exponential model (see Table S4† for lifetime values), which is ascribed to charge transfer from CsFAMA to the SAM and interfaces or surface recombination.^18^*A*_1_ and *A*_2_ are the amplitudes of the respective components, while *τ*_1_ and *τ*_2_ are the lifetimes from the fast and the slow components, respectively. We correlate the *τ*_1_ of the PL decay to carrier transfer processes from the CsFAMA to the SAM. When comparing the PL decay of the samples based on our SAMs, as seen in Fig. 4b, a fast PL decay is observed in contrast to the films with just CsFAMA (*τ*_1_ = 88.83 ns and *τ*_2_ = 874.4 ns). This behaviour suggests that we have efficient hole transfer from the perovskite to the SAM and the lifetimes depending on the structure of the SAMs. Thus, the sequence of the efficiency for the hole transfer is SAM2 (22.65 ns) > SAM1 (23.55 ns) > SAM3 (24.37 ns).

Finally, we have evaluated the photovoltaic performance of the devices prepared with the three SAMs following the architecture ITO/SAM/CsFAMA/PC_61_BM/BCP/Ag.

**Fig. 5:**
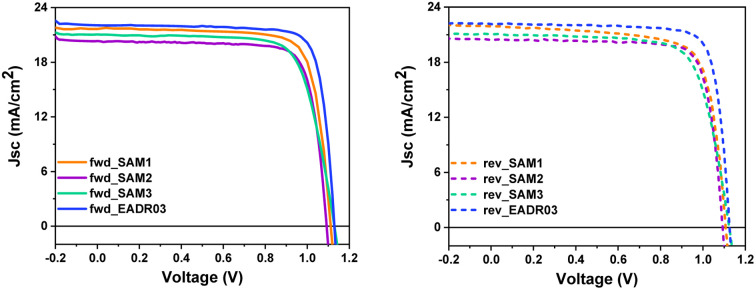
*J*–*V* curves of the champion cells in forward and reverse scan directions for the different SAMs and EADR03.

Fig. 5 displays the current density–voltage (*J*–*V*) curves in forward (fwd) and reverse (rev) scans, whereas Table 1 shows the performance of the champion devices when using SAM1, SAM2, SAM3 and EADR03 as HTMs. The comparison between the forward and reverse scans and the statistics of the performance of the devices are shown in Fig. S4 and Table S5†. On one hand, EADR03 with a champion device performance of 20.18% PCE is comparable to others reported in the literature,^16^ demonstrating that our device procedure is reliable for comparison. SAM efficiencies are very close to the PCE of our reference device, indicating that these new SAMs can be successfully used in iPSCs. The difference in terms of PCE between SAM1 and EADR03 can be attributed to the lower charge transfer observed in the PL decays. On the other hand, the substituent (1,3-dimethoxybenzene) and di-*p*-tolylamine, for the carbazole core in EADR03 and SAM1, respectively, plays a critical role in the electron-donating effect. Unfortunately, the substituent in SAM1 creates undesired consequences such as a steric effect and a deeper LUMO, among others. Regarding the other two SAMs, the addition of a second phenyl group to the linker, as in SAM2, decreases the hysteresis in comparison to SAM1; however there is a loss of 6% in PCE. The substitution of the functional group in SAM3 by di-*p*-tolylamine increases the *J*_sc_ and the *V*_oc_. However, the FF decreases and thus, the overall performance is lower.

**Table 1:** Photovoltaic parameters of the champion devices of the different SAMs and EADR03

HTM	Scan direction	*J*_sc_ (mA cm^−2^)	*V*_oc_ (V)	FF (%)	Efficiency (%)
SAM1	Forward	21.70	1.109	78.3	18.86
	Reverse	21.91	1.105	74.7	18.09
SAM2	Forward	20.32	1.093	79.3	17.61
	Reverse	20.46	1.093	79.9	17.85
SAM3	Forward	21.03	1.130	73.8	17.55
	Reverse	21.04	1.127	73.3	17.40
EADR03	Forward	21.89	1.149	80.24	20.18
	Reverse	21.83	1.144	80.66	20.15

This observation once again highlights the adverse impact of the di-*p*-tolylamine group as a terminal group.

**Fig. 6:**
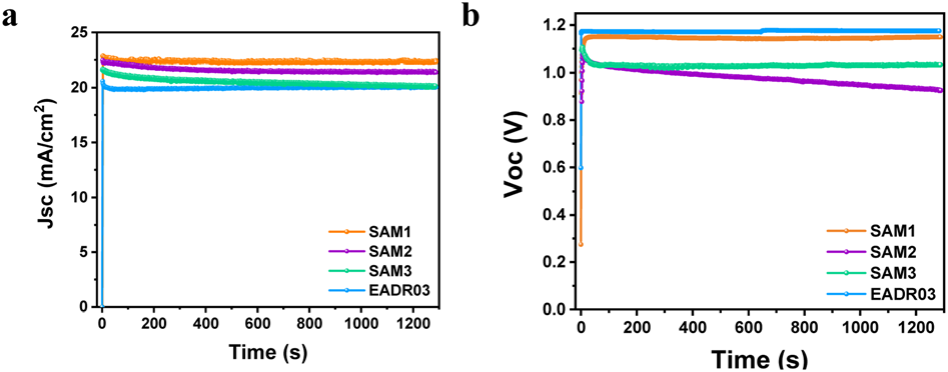
Effect of the long-term continuous illumination of iPSCs based on different SAMs on the (a) *J*_sc_ and (b) *V*_oc_.

The stability of the devices was tested for over 20 minutes under 1 Sun AM1.5G illumination at room temperature, as illustrated in Fig. 6.

Fig. 6a shows an insignificant decrease in *J*_sc_ of 2% for SAM1 and EADR03 and 6% for SAM2 and SAM3 after 20 min. However, the *V*_oc_ of SAM2 decreases from 1.07 V to 0.92 V, while the *V*_oc_ of SAM3 drops from 1.10 V to 1.03 V and then stabilizes. SAM1 displays a stable *V*_oc_ as the reference. To gain additional insights into the effect of SAM1 and EADR03 on device stability, devices having SAM1 and EADR03 were investigated for 100 hours (see Fig. S5†). The *J*_sc_ of the EADR03 based devices decreased to 80% of the initial *J*_sc_ after 94 h. In contrast, the *J*_sc_ of SAM1 gradually decreases to 88% of the initial *J*_sc_ at around 100 h. Significantly, the SAM1 based device shows higher stability with a slowly decreasing trend in *J*_sc_ and *V*_oc_ within 100 h, effectively demonstrating its superior long term illumination stability in comparison to EADR03 in such a period of time.

To better understand the effect of using different SAM structures and the losses in device efficiency, devices with SAM1, SAM2, SAM3 and EADR03 were further analysed under in-operando conditions by using charge extraction (CE) and transient photovoltage (TPV) as advanced optoelectronic techniques. In general, the CE is a technique that quantifies the charge stored in the solar cell under different light intensities and is a valid technique when all kinds of charges are extracted before they recombine.

On the other hand, the TPV technique is utilised to study the carrier recombination process. However, in some cases, the carrier recombination is faster than CE with the same light bias.^18^ In that case, differential capacitance (DC) is an alternative technique to CE, which combines the data from TPV and transient photocurrent (TPV/TPC) measurements.^24^ Fig. S5 and Table S6† show the comparison of the normalized CE and TPV decays under 1 Sun illumination conditions confirming that for all the self-assembled molecules, the CE is a valid technique to evaluate the charge carrier kinetics.

**Fig. 7:**
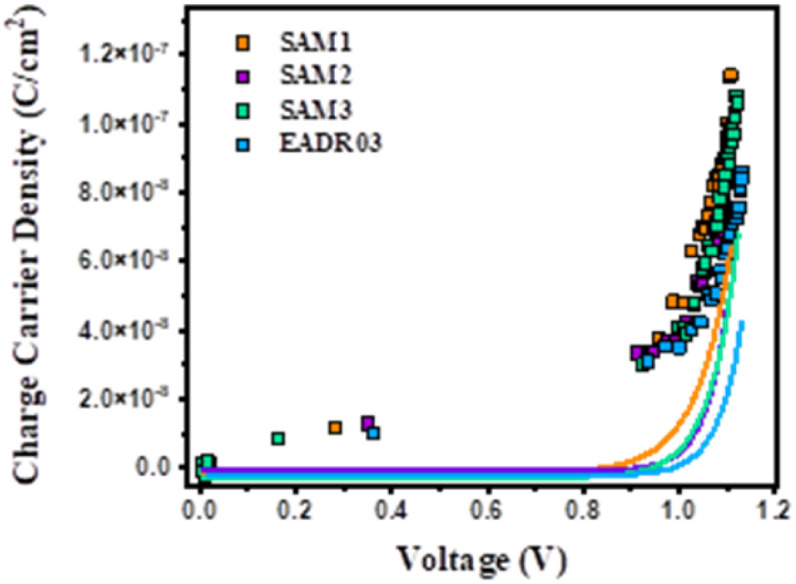
Charge density under different open circuit voltages due to the different illumination conditions with Cgeo and without Cgeo. The solid lines at the bottom are the experimental part of the fits: *y* = BeCx (chemical capacitance) after subtraction of Cgeo (linear part).

Fig. 7 shows the photo-generated charges stored in the cells at equilibrium at different *V*_oc_ values, achieved by tuning the background illumination between 1 sun and the dark. The charge density obtained for the different samples is quite consistent with the JV results observed previously, where SAM1 devices present higher photocurrent than SAM2 and SAM3 devices. Charge density usually exhibited a linear and an exponential part, attributed to the charges accumulated at the interfaces – known as geometrical capacitance, Cgeo – and within the bulk – chemical capacitance. The charges in the bulk (solid lines) present a more pronounced slope at lower voltages for SAM1, SAM2 and SAM3, in comparison to EADR03, meaning that the voltage *vs.* chemical capacitance follows the trend SAM1 < SAM2 ≈ SAM3 < EADR03. These differences in the charge *vs.* voltage indicate changes in the energy offsets with respect to the perovskite valence band (VB). These differences in the charge *vs.* voltage indicate changes in the energy offsets with respect to the perovskite valence band (VB).

**Fig. 8:**
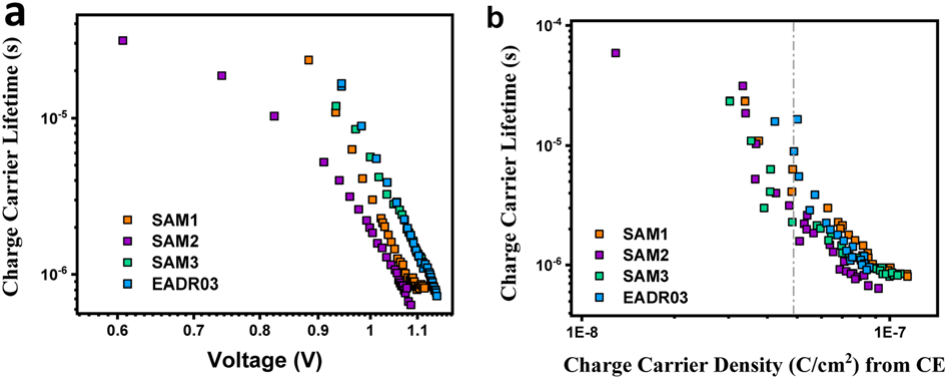
TPV measurements *versus V*_oc_ (a) and *versus* charge density (b) for the different self-assembled molecules.

Next, we analysed the effect the interfacial carrier losses for the different solar cells using TPV measurements. Fig. 8a and b shows the carrier lifetime *versus V*_oc_ and *versus* charge, respectively, for all the samples.

Fig. 8b allows the analysis of the recombination kinetics, since the carrier lifetime directly depends on the charge density, and every SAM presents different photocurrents. The dashed vertical black line is used to compare the differences in carrier lifetime at an equal charge value. The recombination kinetics between the different self-assembled molecules are of the same order of magnitude, but interestingly, SAM2 and EADR03 show faster and slower recombination lifetimes, respectively.

Moreover, it is also possible to obtain the recombination order (*δ*) as *δ* = *λ* + 1,^25^ in which *λ* is obtained by using the following equation:1
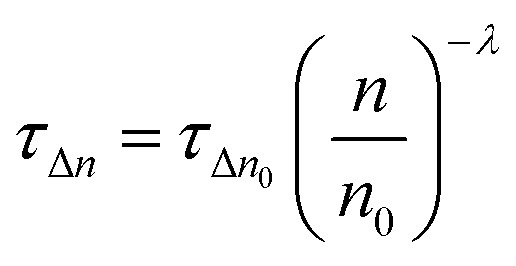


**Fig. 9:**
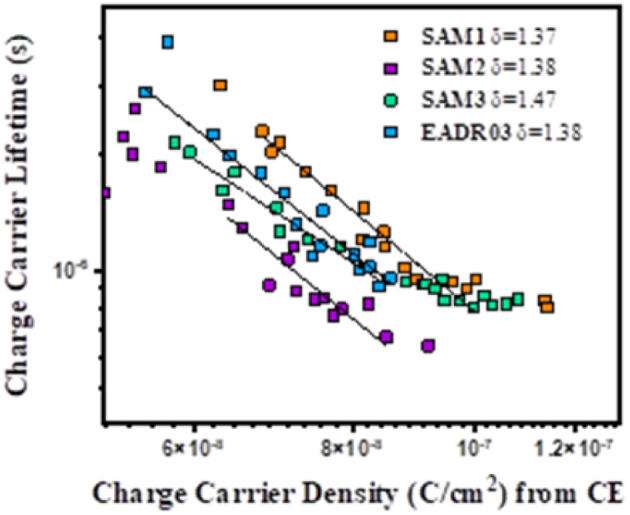
Charge carrier lifetime *versus* charge carrier density indicating the recombination orders.

In eqn (1), *λ* is a parameter that describes the slope of the power law. We obtained the recombination orders of the devices containing different self-assembled molecules: *δ*(SAM1) = 1.37, *δ*(SAM2) = 1.38, *δ*(SAM3) = 1.47, and *δ*(EADR03) = 1.38, see Fig. 9. The deduced recombination orders are very alike and confirm that devices are all ruled by first-order (*δ* = 1), which corresponds to trap-assisted recombination, usually through mid-gap impurities.^26^

## Conclusions

In conclusion, three new molecules have been successfully synthesised and tested in inverted perovskite solar cells with excellent power conversion efficiencies. The different SAMs contained carboxylic acid as an anchoring group, but they were distinguished by phenyl and biphenyl as a linker and three different carbazole core-based functional groups. On one hand, the phenyl group (SAM1) has shown better results in power conversion efficiency and stability in comparison to the biphenyl group (SAM2 and SAM3), indicating that shorter linkers provide better efficiencies for this kind of molecules. On the other hand, the differences of using different terminal groups can be analysed by comparing SAM2 and SAM3, where even if the HOMO energy levels obtained showed significant differences −5.0 and −5.6 eV, respectively, in terms of PCE the results are alike. It is worth noticing that the triphenyl amine group gives rise to smaller grain sizes in the perovskite. Therefore, we can conclude that the linker group in this kind of SAM seems to have a larger effect on the device performance than the functional group. Moreover, SAM1 shows a very good stability, which is comparable to that of the devices with EADR03. The results of this study give important aspects for the future design of new SAMs and the fabrication of efficient and stable iPSCs without the need for chemical dopants in the hole transport layer for future commercialization.

## Author contributions

D. A., E. A., and C. P. synthesised and characterised the SAMs under the supervision of C. P. D. A. and W. L. prepared the devices and measured the performance, while M.M. characterized the photophysical properties. E. M. F. supervised the preparation and characterization of the devices and the analysis of the results. E. P. designed the experiment and analysed the results. All the authors contributed to the discussion of the results and the writing of the manuscript.

## Conflicts of interest

There are no conflicts to declare.

## Supplementary Material

NA-005-D3NA00811H-s001
