# Correction: Influence of the carbazole moiety in self-assembling molecules as selective contacts in perovskite solar cells: interfacial charge transfer kinetics and solar-to-energy efficiency effects

**DOI:** 10.1039/d4na90051k

**Published:** 2024-05-03

**Authors:** Dora A. González, Carlos E. Puerto Galvis, Wenhui Li, Maria Méndez, Ece Aktas, Eugenia Martínez-Ferrero, Emilio Palomares

**Affiliations:** a Institute of Chemical Research of Catalonia (ICIQ-CERCA) Avda. Països Catalans 16 Tarragona Spain epalomares@iciq.es; b Department of Electric, Electronic and Automatic Engineering, Universitat Rovira i Virgili Avda. Països Catalans 26 Tarragona Spain; c ICREA Passeig Lluís Companys 23 Barcelona Spain

## Abstract

Correction for ‘Influence of the carbazole moiety in self-assembling molecules as selective contacts in perovskite solar cells: interfacial charge transfer kinetics and solar-to-energy efficiency effects’ by Dora A. González *et al.*, *Nanoscale Adv.*, 2023, **5**, 6542–6547, https://doi.org/10.1039/d3na00811h.

The authors regret that in the original manuscript, Fig. 4b and c contain errors in the units of the *X*-axis. The *X*-axis was incorrectly labelled as Time (μs) instead of Time (ns). The authors confirm that the results and conclusions of the original manuscript are unaffected by this error. The correct Fig. 4 is given below.

**Fig. 4:**
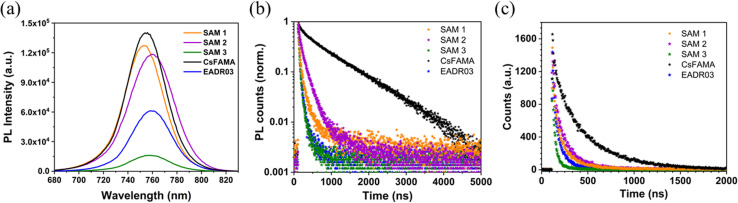
(a) Steady-state photoluminescence spectra and (b) normalized time-resolved photoluminescence decays with a fixed 5000 acquisition counts and (c) with a fixed time at 300 seconds. The samples were excited from the glass side (635 nm) at 770 nm.

The Royal Society of Chemistry apologises for these errors and any consequent inconvenience to authors and readers.

## Supplementary Material

