# Supplementary material for: Influence of the carbazole moiety in self-assembling molecules as selective contacts in perovskite solar cells: interfacial charge transfer kinetics and solar-to-energy efficiency effects
Source: Nanoscale Adv. 2023 Oct 16;5(23):6542–7. doi: 10.1039/d3na00811h (PMC10662120; doi:10.1039/d3na00811h)
Supplement: NA-005-D3NA00811H-s001 [file NA-005-D3NA00811H-s001.pdf]

Electronic Supplementary Material (ESI) for Nanoscale Advances.
This journal is © The Royal Society of Chemistry 2023

Supporting Information

Influence of the Carbazole Moiety in Self-Assembling Molecules as Selective Contacts in Perovskite Solar Cells: Interfacial Charge Transfer Kinetics and Solar-to-Energy Efficiency Effects.

Dora A. González[1,2], Carlos E. Puerto Galvis[1], Wenhui Li[1], Maria Mendéz[1], Ece Aktas[1,3], Eugenia Martínez-Ferrero[1] and Emilio Palomares,[1,4]*

[1]*Institute of Chemical Research of Catalonia (ICIQ). Avda. Paÿsos Catalans, 16. Tarragona. E-43007. Spain.*

[2]*Departmant d'Enginyeria Electrònica, Eèctrica I Automàtica. URV. Tarragona. E-43007. Spain.*

[3] *Current address: Department of Chemical, Materials and Production Engineering, University of Naples Federico II, Piazzale Tecchio 80, 80125 Fuorigrotta, Italy*

[4]*ICREA. Passeig Lluís Companys 23. E-08010. Spain.*

Email of corresponding authors:

*epalomares@ICIQ.es

Contents

EXPERIMENTAL PROCEDURES 3

MATERIALS 3
METHODS 3
DEVICE PREPARATION 3

CHARACTERIZATION 5

MEASUREMENTS OF THE SAMS: 5
2.1.1 Techniques *5*
2.1.2 Tables and Figures *5*
FILM AND DEVICE CHARACTERIZATION 6
2.2.1 Techniques *6*
2.2.2 Tables and Figures *7*

SYNTHESIS 12

SYNTHESIS OF SAM1 12
Synthesis of SAM 2 *15*
Synthesis of SAM 3 *19*
^{1}H and ^{13}C spectra *21*
Mass Spectrum Spectra *31*

REFERENCES 32

EXPERIMENTAL PROCEDURES

Materials

All the chemical materials and solvents were purchased from Sigma-Aldrich, TCI Europe, MERCK, Fluorochem, Dyenamo, Lumtec, Apollo Scientific, Emplura, Panreac or Alfa Aesar and they were used without further purification unless otherwise mentioned. Common solvents are purchased from SdS.

The patterned indium tin oxide (ITO) glass substrates (1.5 cm × 1.5 cm, 15 Ω sq^{-1}) were purchased from Xin Yan Technology Ltd.

Methods

All sensitive reactions were carried out under an argon atmosphere using standard Schlenk techniques (under high-vacuum, ~10^{-2} mbar) with distilled and dried solvents unless otherwise stated. The course of the reactions was checked by thin layer chromatography (TLC) using Merck silica gel 60 F_{254} precoated plates (0.25 mm) and visualised with a UV lamp. All workup and purification procedures were carried out with reagents and solvents with analytical grade. All the solvents were removed by rotary evaporation under reduced pressure. Colum chromatography used spherical silica gel 70 Å, 40–75 mm.

Device preparation

Patterned ITO substrates were cleaned sequentially for 15 min with ethanol and IPA in an ultrasonic bath, then dried with N_2 gas. Before using, the substrates are treated with ultraviolet ozone (UV-O_3) for 30 minutes. Finally, the ITOs were transferred to the globe box to complete the device.

The SAM layer was applied by one step on the treated ITO by spin-coating without thermal annealing. The spin coating sequence was at 3000 rpm for 30 s with an acceleration of 2000 rpm s^{-1} and the concentration of the SAM solution was optimised to 0.1 M in THF anhydrous.

The CsFAMA perovskite solution was prepared as reported in the literature.[1,2] The precursor solution of $(Cs_{0.05}(FA_{0.85}MA_{0.15})_{0.95}Pb(I_{0.85}Br_{0.15})_3$ was made by mixing FAI (1.1 M), PbI_2 (1.15 M), MABr (0.2 M) and $PbBr_2$ dissolved in anhydrous DMF:DMSO (4:1 volume ratio). Finally, 42 µL of CsI solution (1.5 in DMSO) was added to obtain the CsFAMA perovskite solution. The CsFAMA solution was deposited onto the SAMs by spin-coating with two-step deposition method; 1) 80 µL of CsFAMA at 2000 rpm for 10

s with an acceleration of 2000 rpm s^{-1} and 2) 4000 rpm for 25s, where 100 µL of chlorobenzene (CBZ) was dispensed on the spinning substrate in the last 12 s. Then, thermal annealing at 100 ºC for 40 min was immediately applied.

Afterwards, 70 µL of a $PC_{61}BM$ solution (20 mg/mL) in 1 mL of CBZ was dynamically deposited by one-step method at 1000 rpm for 1 min with an acceleration of 1000 rpm s^{-1} on top of the perovskite layers. Then, a solution of BCP (1 mg·mL^{-1}) in IPA was spin-coated at 4000 rpm for 30 s with an acceleration of 2000 rpm s^{-1}.

Finally, Ag (100 nm) was evaporated under a high vacuum ($1x10^{-6}$ bar) as a counter electrode, defining the active area of the devices at 0.09 cm^2.

CHARACTERIZATION

Measurements of the SAMs:

2.1.1 Techniques

Ultraviolet-visible (UV-vis) absorption spectroscopy was performed to measure the absorbance of SAMs in solution. UV-vis was measured using Shimadzu UV spectrophotometer 1700 with optical range from 190 and 1000 nm.

Steady-state fluorescence emission (PL) spectra were recorded on Horiba Jobin Yvon Lrd. Width a PMT (UV-vis) and InGaAs (NIR) detectors with wavelengths ranging from 250 to 1600 nm. All samples were measured using 1 cm path-length quartz cell at room temperature with a concentration of ~10^{-5} M in dichloromethane (DCM).

Thermogravimetric analysis (TGA) technique was chosen to determine the weight loss of a material as a function of temperature or time under a controlled atmosphere. The decomposition temperature (Tdes) was investigated from 5% weight loss at a scan rate of 10 ºC/min under a N2 atmosphere, where the working temperature went from 30 to 1000 ºC. The measurements were carried out in a TGA/SDTA851 Mettler Toledo equipment.

From differential scanning calorimetry (DSC) analysis it was possible to observe information about physical and chemical changes such as melting (Tm), glass transition (Tg), and crystallisation (Tc) temperatures. DSC measurements were recorded on DSC822e from Mettler Toledo instrument with a heating rate of 10 °C/min under N_2 atmosphere by heating from 30 to 650 ºC.

2.1.2 Tables and Figures

Table S1. Thermal and optical properties of SAMs

	T_{dec} (ºC)[(a)]	λ_{abs} (nm)	λ_{onset} (nm)	E_g^{opt} (b)
SAM1	246	299	414	2.99
SAM2	273	304	393	3.13
SAM3	274	320	369	3.36

(a) Decomposition temperature determined from TGA (5 % weight loss) and the experiment was carried out under N_2 atmosphere, scan rate of 10 °C/min. (b) Optical band gap (E_g^{opt}) estimated from the edge of absorption spectra

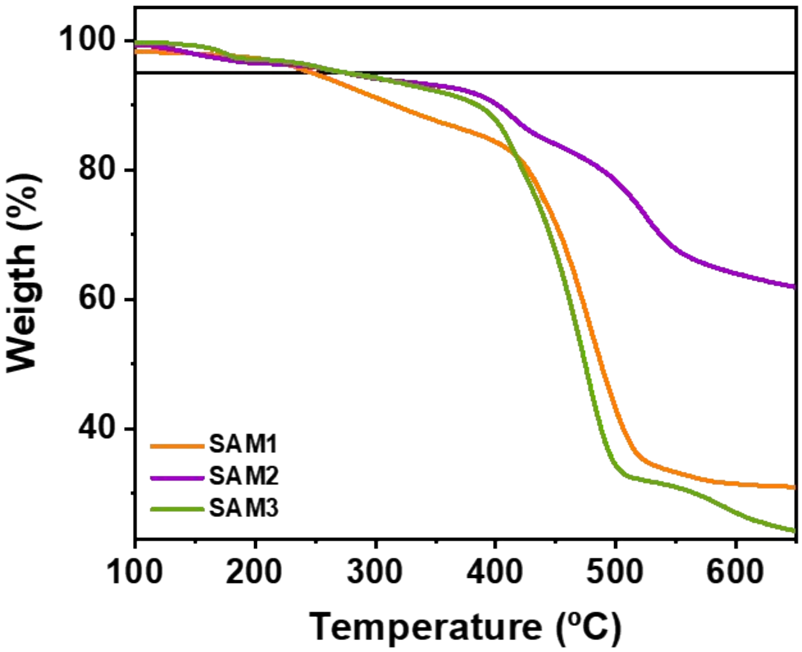

Figure S1. TGA of SAM molecules at a scan rate of 10 °C/min under N_2 atmosphere.

Film and Device Characterization

2.2.1 Techniques

Contact angle measurements were carried out by Attention Theta Flex optical tensiometer, using sessile water drop analysis.

The field-emission scanning electron microscopy (FESEM) was carried out by using FEI Quanta 600 microscopy to obtain the surface morphology of perovskite on top of the SAMs or bare ITO. All perovskite films were protected with PMMA to do measurements under ambient conditions. The mean values of grain size for perovskite films were obtained by calculating the sizes of 50 grains using the ImageJ program.

The X-ray Photoelectron Spectroscopy (XPS) was used to study the chemical state of the atoms of the SAM molecules on the treated ITO. XPS measurements were performed at room temperature with a SPECS PHOIBOS 150 hemispherical analyser (SPECS GmbH, Berlin, Germany)) in a base pressure of 5x10-10 mbar. For XPS we were using monochromatic Al K alpha radiation (1486.74 eV) as an excitation source operated at 300 W. The energy resolution as measured by the FWHM of the Ag 3d5/2 peak for a sputtered silver foil was 0.62 eV. For UPS we were using a monochromatic HeI line at 21.2 eV.

Steady-state photoluminescence (PL) and time-resolved photoluminescence (TRPL) spectra were performed on Edinburgh Instruments LifeSpec-II spectrometer using the single photon counting technique with a 635 nm laser excitation under ambient conditions. All perovskite films were protected with PMMA.

The current-voltage (*J-V*) curves were recorded using a Solar Simulator (ABET 11000) and a source meter (Keithley 2400). The curves were registered under 1 Sun conditions (100 mW/cm^2, AM 1.5G) calibrated with a silicon reference cell. The scan rate employed was 80 mV/s.

Long-term illumination stability of the unencapsulated devices was performed in sealed holder filled with nitrogen under 1) 1 Sun conditions with a Solar Simulator (same condition as *J-V* curves), and 2) under continuous white-light LED at room temperature. The light LED intensity was calibrated to achieve the same J_{sc} from the iPSCs under standard 1 Sun AM1.5G illumination and the photovoltage measurements were recorded 60 min by the software.

Photo-induced charge extraction (CE), transient photovoltage (TPV) and transient photocurrent (TPC) measurements were carried out using a white LED controlled by a programmable power supply and box that switches from open to short-circuit states. All the signals are recorded using a Yokogawa DLM2052 oscilloscope, registering drops in voltage. Light perturbation pulses for TPV and TPC were provided by nanosecond PTI GL-3300 nitrogen laser and using a 550 nm laser pulse wavelength.

2.2.2 Tables and Figures

Table S2. Average grain size in perovskite films determined from FESEM images.

	Mean	
**Sample**	**Length (nm)**	**Area (nm^2)**
ITO/SAM1/CsFAMA	289	61
ITO/SAM2/CsFAMA	269	57.
ITO/SAM3/CsFAMA	312	69
ITO/CsFAMA	306	66

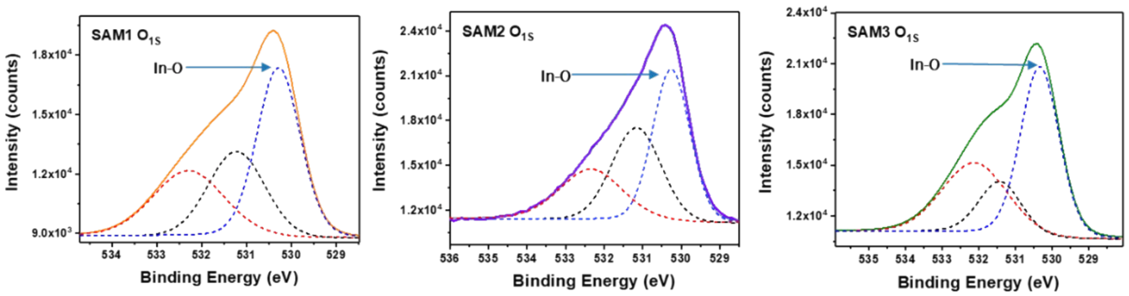

Figure S2. The XPS high resolution survey spectra of O1s of the different SAMs used in this work.

Table S3. The functional groups and their related binding energies extracted from XPS spectra of SAM1, SAM2 and SAM3.

	C1s (eV)		O1s (eV)		N1s (eV)
Samples	C-C/C-H	O=C-O	O_2^-	C=O	C-N
ITO/SAM1	286.1	288.8	531.2	532.3	400.5
ITO/SAM2	285.9	288.5	531.1	532.4	400.3
ITO/SAM3	285.5	288.9	531.5	532.1	400.5
ITO	286.3	289.0	532.1	536.5	-

Table S4. Decay fitting parameters determined from the TRPL curves.

**Samples**	**A_1**	**τ_1 (ns)**	**A_2**	**τ_2 (ns)**
ITO/SAM1/CsFAMA	1.13	23.55	0.17	132.38
ITO/SAM2/CsFAMA	0.54	22.65	0.53	151.44
ITO/SAM3/CsFAMA	1.12	24.37	0.04	151.40
ITO/CsFAMA	1.12	88.83	0.74	874.40
ITO/EADR03/CsFAMA	1.15	29.48	0.03	214.31

[a] Counts collected within a fixed time of 300 s.

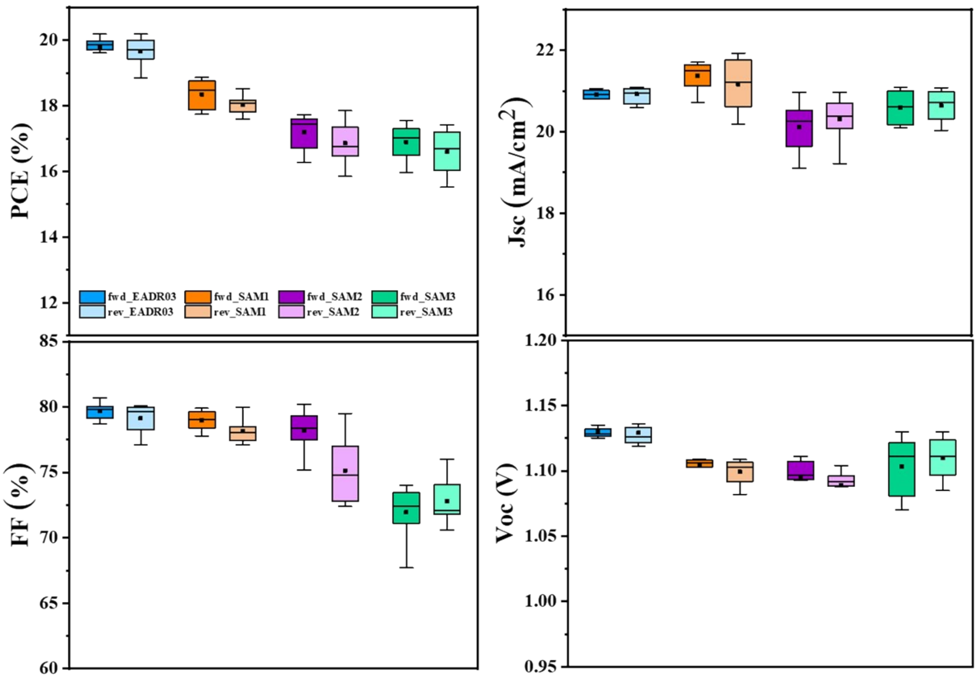

Figure S3. Comparison of cell performance statistics with all the SAMs.

Table S5. The photovoltaic parameter statistics of an average of 8 devices based on SAM1, SAM2, SAM3 and EADR03.

HTM	Scan direction	Jsc (mA/cm^2)	Voc (V)	FF (%)	Efficiency (%)
SAM 1	fwd	21.37 ± 0.37	1.10 ± 0.005	79.0 ± 0.7	18.35 ± 0.46
	rev	21.16 ± 0.68	1.09 ± 0.009	78.1 ± 0.9	18.02 ± 0.28
SAM 2	fwd	20.11 ± 0.63	1.09 ± 0.025	78.2 ± 1.6	17.19 ± 0.55
	rev	20.31 ± 0.56	1.09 ± 0.015	75.1 ± 2.9	16.86 ± 0.67
SAM 3	fwd	20.59 ± 0.42	1.10 ± 0.023	71.9 ± 2.0	16.89 ± 0.55
	rev	20.64 ± 0.40	1.10 ± 0.016	72.8 ± 1.7	16.59 ± 0.69
EADR03	fwd	20.91 ± 0.47	1.13 ± 0.006	79.67 ± 1.7	19.02 ± 0.56
	rev	20.92 ± 0.54	1.13 ± 0.011	79.12 ± 1.1	19.00 ± 0.43

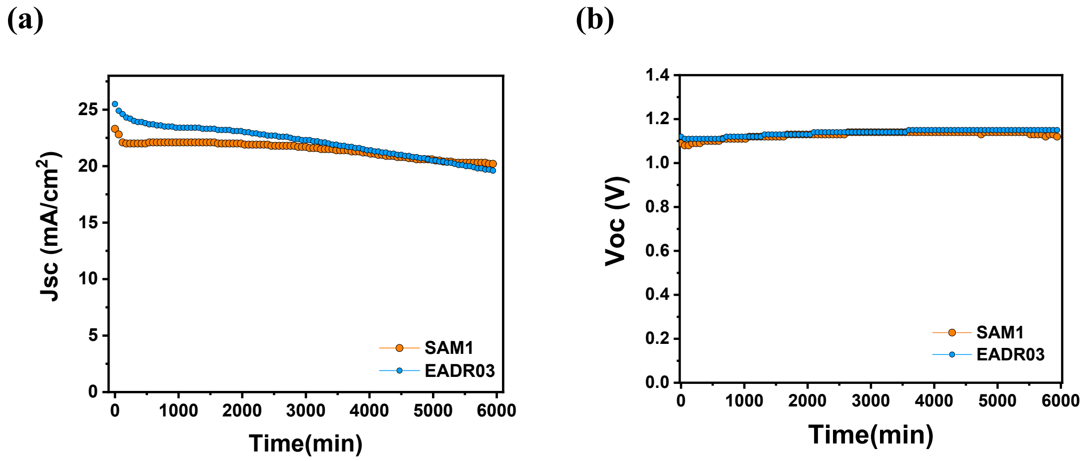

Figure S4. Long-term continuous illumination of iPSCs based on **SAM1** and EADR03 (**a**) Jsc and (**b**) Voc, using a Solar Simulator under standard 1 Sun AM1.5G.

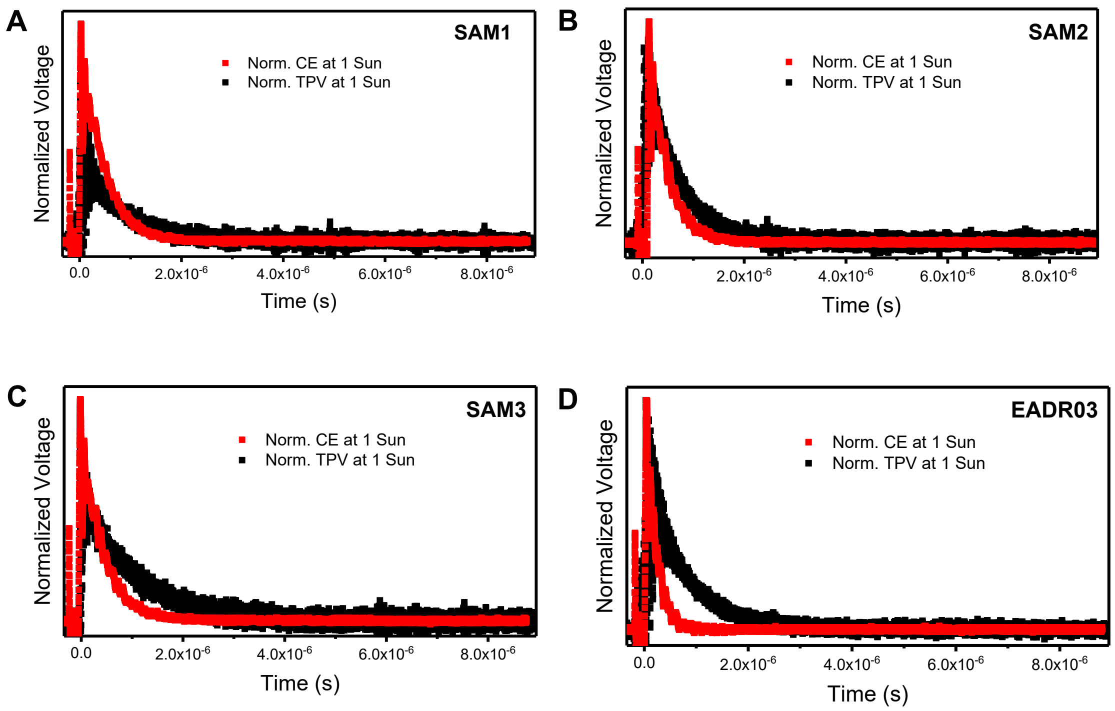

Figure S5. The CE and TPV decays under 1 Sun illumination conditions for **SAM1** (**A**), **SAM2** (**B**), **SAM3** (**C**) and EADR03 (**D**).

Table S6. CE and TPV decays lifetime at 1 Sun illumination conditions. Two diodes of every molecule are shown, indicating reproducible results.

	**CE (μs)**	**TPV (μs)**
SAM1	0.497	0.82
SAM2	0.353	0.493
SAM3	0.447	1.055
EADR03	0.226	0.785

SYNTHESIS

Synthesis of SAM1

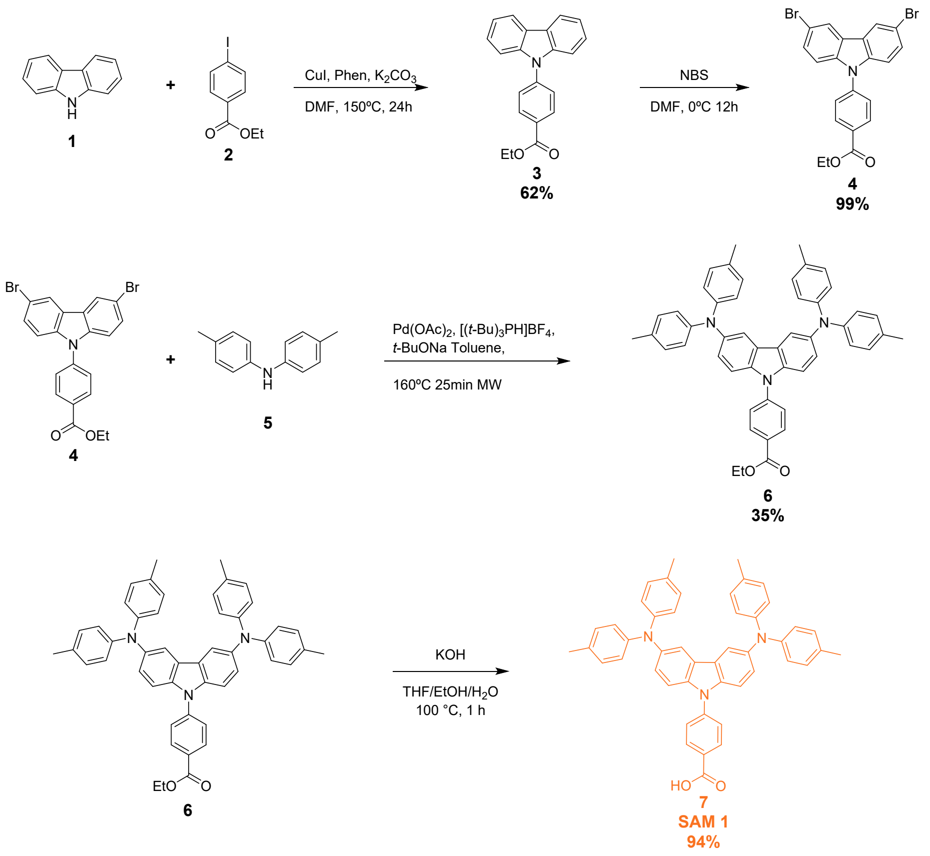

Scheme S1 Synthetic pathway for the synthesis of SAM 1

Synthesis of ethyl 4-(9*H*-carbazol-9-yl)benzoate (3)

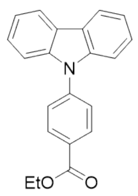

3

Procedure 1a: A solution of CuI (160 mg, 0.83 mmol), 1, 10-Phenanthroline (150 mg, 0.83 mmol), K_2CO_3 (1.73 g, 12.55 mmol) and ethyl 4-iodobenzoate (**2**) (2.50 g, 9.21 mmol) was dissolved in dry DMF (15 mL). The mixture was stirred for one under argon atmosphere. Then, 9*H*-carbazole (**1**) (1.40 mg, 8.37 mmol) was added and the reaction mixture was stirred at 150 ºC for 24 h. After cooling down, the solution was filtered through celite to remove insoluble solids and concentrated under reduced pressure. The

resulting solid was purified by silica gel column chromatography to obtain **3** as a white powder (1.61 g, 62%). **^{1}H NMR** (500 MHz, $CDCl_3$) $\delta_{(ppm)}$: 8.30 (2H, d, *J* = 8.5 Hz, 3 and 16-H_{Ar}), 8.15 (2H, d, *J* = 7.8 Hz, 7 and 14-H_{Ar}), 7.69 (2H, d, *J* = 8.5 Hz, 4 and 15-H_{Ar}), 7.51 – 7.41 (4H, m, 8, 10, 11 and 13-H_{Ar}), 7.32 (2H, t, *J* = 7.4 Hz, 9 and 12-H_{Ar}), 4.46 (2H q, *J* = 7.2 Hz, OCH_2), 1.46 (3H t, *J* = 7.1 Hz, -CH_3). **^{13}C NMR** (126 MHz, $CDCl_3$) $\delta_{(ppm)}$: 165.9, 141.9, 140.3, 131.3, 129.1(2C), 126.4(2C), 126.2(2C), 123.8(2C), 120.5(2C), 120.5(2C), 120.5(2C), 109.8(2C), 61.3, 14.4.

Synthesis of ethyl 4-(3,6-dibromo-9H-carbazol-9-yl)benzoate (4)

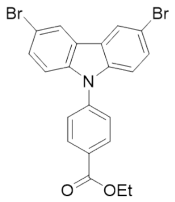

4

Procedure 1b: A solution of NBS (1.73 g, 9.75 mmol) in 7 mL DMF was added dropwise into a solution of **3** (0.7 g, 2.32 mmol) in DMF (20 mL). The solution was shielded from light and stirred for12 h at 0 ºC. The reaction mixture was quenched with crushed ice and the formed precipitated was filtered, washed with water and dried to gave **4** as a white product (1.06 g, >99). **^{1}H NMR** (500 MHz, $CDCl_3$) $\delta_{(ppm)}$: 8.30 (2H, d, *J* = 8.5 Hz, 3 and 16-H_{Ar}), 8.20 (2H,d, *J* = 2.0 Hz, 10 and 11-H_{Ar}), 7.60 (2H, d, *J* = 8.6 Hz, 4 and 15-H_{Ar}), 7.52 (2H, dd, *J* = 8.7, 1.9 Hz, 7 and 14-H_{Ar}), 7.30 (2H, d, *J* = 8.7 Hz, 8 and 13-H_{Ar}), 4.46 (2H, q, *J* = 7.1 Hz, OCH_2), 1.45 (3H, t, *J* = 7.1 Hz, CH_3). **^{13}C NMR** (126 MHz, $CDCl_3$) $\delta_{(pmm)}$: 166.0, 142.0, 140.3 (2C), 131.4, 129.1 (2C), 126.4 (2C), 126.2 (2C), 123.8 (2C), 120.5 (2C), 120.5 (2C), 109.8 (2C), 61.3, 14.4.

Synthesis of Ethyl 4-(3,6-bis(di-p-tolylamino)-9*H*-carbazol-9-yl)benzoate (6)

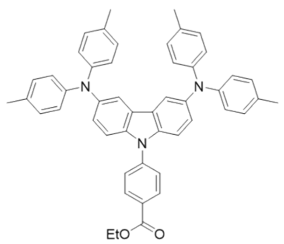

6

Procedure 1c: In the presence of argon atmosphere, a solution of **5** (0.35 g, 1.77 mmol), $Pd(OAc)_2$ (38 mg, 0.164 mmol), [(*t*-Bu)$_3$PH]BF_4 (TTBP) (69 mg, 0.338 mmol) and *t*-BuONa (0.28 g, 3 mmol) were dissolved in anhydrous toluene (10mL). After 30 min, **4**

was added in one portion. The solution was heated under MW irradiation for 25 min at 160 ºC. The reaction crude was filtered through celite and the solvent was removed under reduced pressure. Finally, the crude product was purified using silica gel column chromatography (HEX:EtOAc, 20:1, v//v) to obtain a pale-yellow solid (0.21 g 35%). **^{1}H NMR** (500 MHz, THF) $\delta_{(ppm)}$: 8.27 (2H, d, J = 8.8 Hz, 1 and 26-H_{Ar}), 7.77 – 7.74 (4H, m, 3, 4, 23 and 24-H_{Ar}), 7.40 (1H, s, 13-H_{Ar}), 7.38 (1H, s, 14-H_{Ar}), 7.14 (2H, dd, J = 8.8, 2.2 Hz, 2 and 25-H_{Ar}), 6.97 (8H, d, J = 8.1 Hz, 5, 8, 9, 12, 15, 18, 19, and 22-H_{Ar}), 6.89 (8H, d, J = 8.5 Hz, 6, 7, 10, 11, 16, 17, 20, and 21-H_{Ar}), 4.39 (2H, q, J = 7.1 Hz, OCH_2), 2.23 (12H, s, -CH_3), 1.40 (3H, t, J = 7.1 Hz, -CH_3). **^{13}C NMR** (101 MHz, THF) $\delta_{(ppm)}$: 166.1, 147.6 (4C), 143.0, 142.9 (2C), 138.6 (2C), 132.2 (8C), 131.8 (4), 130.5, 127.2, 126.5 (2C), 125.8 (2C), 124.0 (2C), 123.8 (8C), 119.0 (2C), 111.6 (2C), 61.8, 20.9 (4C), 14.8.

Synthesis of 4-(3,6-bis(di-p-tolylamino)-9*H*-carbazol-9-yl)benzoic acid (SAM 1)

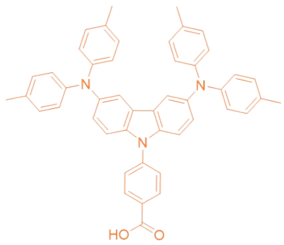

7

Procedure 1d: A solution of compound **6** (0.2 g, 0.289 mmol) in THF (6 mL) was added in one portion to a solution of KOH (48.6 mg, 0.86 mmol) in EtOH:H_2O (1mL, 1:1, v/v). The mixture was refluxed at 100 ºC for 1 hour. After cooling to room temperature, the mixture was extracted with ethyl acetate (2 x 100 mL). Then, 20 mL of HCl solution (5 M) were added to neutralize the mixture. The combined organic layers were filtered and concentrated under reduced pressure. The final product was obtained via crystallization from hexane as light-green solid (0.184 g, 94%). **SAM1** was characterized by ^{1}H and ^{13}C NMR spectrometry as well by HRMS **^{1}H NMR** (400 MHz, Acetone) $\delta_{(ppm)}$: 8.31 (2H, d, J = 8.6 Hz, 1 and 26-H_{Ar}), 8.02 (1H, s, -OH), 7.84 (2H, d, J = 8.6 Hz, 2 and 25-H_{Ar}), 7.80 (2H, d, J = 2.3 Hz, 13 and 14-H_{Ar}), 7.49 (2H, d, J = 8.8 Hz, 4 and 23-H_{Ar}), 7.20 (2H, dd, J = 8.8, 2.2 Hz, 3 and 24-H_{Ar}), 7.04 (8H, d, J = 8.4 Hz, 5, 8, 9, 12, 15, 18, 19, and 22-H_{Ar}), 6.91 (8H, d, J = 8.5 Hz, 6, 7, 10, 11, 16, 17, 20, and 21-H_{Ar}), 2.25 (12H, s, -CH_3). **^{13}C NMR** (101 MHz, Acetone) $\delta_{(ppm)}$: 165.7, 146.4 (5C), 141.9 (2C), 137.3 (2C), 131.3 (2C), 131.1 (3C), 129.7 (8C), 126.2 (4C), 125.3 (2C), 124.5 (2C), 122.9 (8C), 117.5 (2C),

110.8 (2C), 19.8 (4C). m/z calculated for $C_{47}H_{39}N_3O_2$ [M^+] exact mass= 677.3042, found MS (MALDI-TOF) = 677.3041.

Synthesis of SAM 2

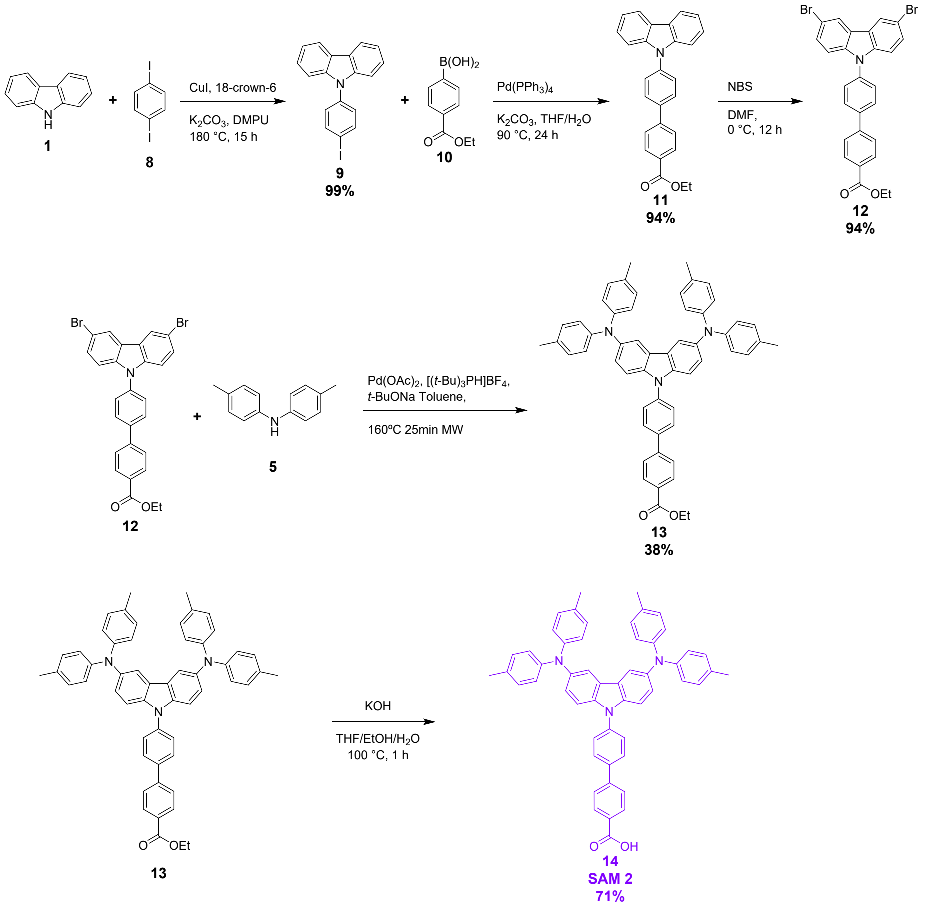

Scheme S2 Pathway of SAM 2

Synthesis of 9-(4-iodophenyl)-9H-carbazole (9)

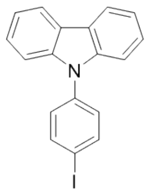

9

Procedure 2a: To a 50 mL vial charged with a stir bar was added CuI (28 mg, 0.14 mmol), 18-crown-6 (13 mg, 0.04 mmol), carbazole **1** (500 mg, 2.99 mmol) and K_2CO_3 (826 mg, 5.97 mmol) were dissolved in 20 mL of **DMPU** under Ar atmosphere. The system was stirred for one hour at room temperature and 1,4-diiodobenzene (1.99 g, 5.98 mmol) was added. The reaction mixture was then heated at 140 ºC for another hour and then at 180 ºC for 12 h. The reaction crude was filtered through celite and the solvent was

removed under reduced pressure. The residue was purified by column chromatography on silica gel with HEX:EtOAc (20:1, v/v) as eluent to obtain compound **9** as a light-yellow solid (1.09 g, 99%). **^{1}H NMR** (500 MHz, $CDCl_3$) $\delta_{(ppm)}$: 8.15 (2H d, J = 7.7 Hz,6 and 7-H_{Ar}), 7.93 (2H d, J = 8.6 Hz, 1and 2-H_{Ar}), 7.45 – 7.37 (4H, m, 2, 3, 4, and 5-H_{Ar}), 7.37 – 7.28 (4H, m, 8, 9, 10 and 11-H_{Ar}). **^{13}C NMR** (126 MHz, $CDCl_3$) $\delta_{(ppm)}$: 140.7, 139.2 (2C), 137.7, 129.1 (2C), 126.2 (2C), 123.7 (2C), 120.5 (2C), 120.4 (2C), 109.7 (2C), 92.2.

Synthesis of Ethyl 4'-(9H-carbazol-9-yl)-[1,1'-biphenyl]-4-carboxylate (11)

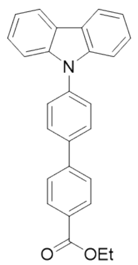

11

Procedure 2b: A mixture of **9** (0.462 g, 1.25 mmol), K_2CO_3 (0.346 g, 2.5 mmol), Pd((PPh$_3$)$_4$ (58 mg, 0.005 mmol) and compound **10** (0.315 g, 1.58 mmol) were dissolved in 9 mL of THF:H_2O (2:1) and the reaction mixture was stirred at 90 ºC for 24 hours. The reaction mixture was cooled down to room temperature and subsequently was extracted by EtOAc (3 x 50 mL), washed with a saturated brine solution (3 x 15 mL), dried over anhydrous Na_2SO_4 and filtered. The organic mixture was evaporated under reduced pressure and the residue was purified by silica-gel column chromatography using Hexane and EtOAc as eluents (20:1, v:v) to obtain a 0.452 g of **11** (94%). **^{1}H NMR** (400 MHz, $CDCl_3$) $\delta_{(ppm)}$: 8.21 – 8.15 (4H, m, 1, 8, 9 and 16-H_{Ar}), 7.86 (2H, d, J = 8.6 Hz, 5 and 12-H_{Ar}), 7.77 (2H, d, J = 8.6 Hz, 4 and 13-H_{Ar}), 7.68 (2H, d, J = 8.6 Hz, 3 and 14-H_{Ar}), 7.51 – 7.41 (4H, m, 6, 7, 10 and 11-H_{Ar}), 7.35 – 7.29 (2H, m, 2 and 15-H_{Ar}), 4.44 (2H, q, J = 7.1 Hz, OCH_2), 1.45 (3H, t, J = 7.1 Hz, -CH_3). **^{13}C NMR** (101 MHz, $CDCl_3$) $\delta_{(ppm)}$: 166.6, 144.6, 140.9, 139.2 (2C), 137.8, 130.4, 129.8 (2C), 128.8 (2C), 127.5(2C), 127.1 (2C), 126.2 (2C), 123.7 (2C), 120.5 (2C), 120.3 (2C), 109.9 (2C), 61.2, 14.5.

Ethyl 4'-(3,6-dibromo-9H-carbazol-9-yl)-[1,1'-biphenyl]-4-carboxylate (12)

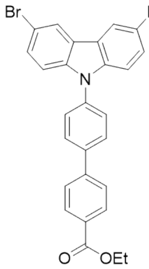

12

Following the procedure **1b**, compound **12** was obtained as a white solid (0.72 g, 94%). ^{1}**H NMR** (500 MHz, $CDCl_3$) $\delta_{(ppm)}$: 8.22 – 8.15 (4H, m, 1, 7, 8 and 14-H_{Ar}), 7.85 (2H, d, J = 7.7 Hz, 3 and 12-H_{Ar}), 7.74 (2H, d, J = 8.7 Hz, 2 and 13-H_{Ar}), 7.59 (2H, d, J = 8.7 Hz, 5 and 10-H_{Ar}), 7.52 (2H, dd, J = 8.7, 1.9 Hz, 4 and 11-H_{Ar}), 7.31 (2H, d, J = 8.7 Hz, 6 and 9-H_{Ar}), 4.43 (2H, q, J = 7.1 Hz, -OCH_2), 1.44 (3H, t, J = 7.1 Hz, -CH_3). ^{13}**C NMR** (126 MHz, $CDCl_3$) $\delta_{(ppm)}$: 173.2, 166.5, 144.3, 139.9 (2C), 136.8, 130.4, 129.9 (2C), 129.6 (2C), 129.1 (2C), 127.4 (2C), 127.1 (2C), 124.2 (2C), 123.4 (2C), 113.4 (2C), 111.6 (2C), 61.3, 14.5.

Ethyl 4'-(3,6-bis(di-p-tolylamino)-9H-carbazol-9-yl)-[1,1'-biphenyl]-4-carboxylate (13)

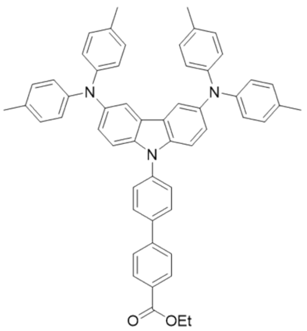

13

Following procedure **1c**, crude product **13** was obtained and purified by column chromatography (HEX:EtOAc, 15:1) to obtain 67 mg (38%) of pale green solid. However, due to the poor solubility of **13**, it was not possible to confirm its structure by NMR. Therefore, the reduction was carried out to obtain **SAM 2** following the procedure **1d**.

4'-(3,6-bis(di-p-tolylamino)-9H-carbazol-9-yl)-[1,1'-biphenyl]-4-carboxylic acid (SAM 2)

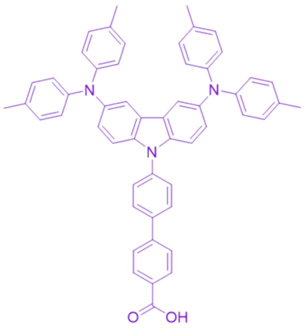

14
SAM 2

SAM 2 was synthesized following procedure **1d** to give a light-green solid (231 mg, 92%). **SAM 2** was characterized by ^{1}H and ^{13}C NMR spectrometry as well by HRMS (Annex X) **^{1}H NMR** (400 MHz, Acetone) $\delta_{(ppm)}$: 8.21 – 8.14 (2H, m, 1 and 30-H_{Ar}), 8.07 (2H, d, *J* = 8.7, 2.3 Hz, 5 and 26-H_{Ar}), 7.95 (2H, d, *J* = 8.6 Hz, 16 and 15-H_{Ar}), 7.80 (4H, dd, *J* = 7.1, 1.7 Hz, 4, 5, 26 and 27-H_{Ar}), 7.45 (2H, d, *J* = 2.5 Hz, 6 and 25-H_{Ar}), 7.21 (2H, ddd, 3.7 Hz, 3 and 28-H_{Ar}), 7.04 (8H, d, *J* = 8.3 Hz, 7, 10, 11, 14, 17, 20, 21 and 24-H_{Ar}), 6.91 (8H, d, *J* = 8.5 Hz, 8, 9, 12, 13, 18, 19, 22 and 23-H_{Ar}), 2.25 (12H, s, -CH_3). **^{13}C NMR** (101 MHz, Acetone) $\delta_{(ppm)}$: 147.5 (3C), 142.5, 138.9, 132.0 (4C), 131.4 (2C), 131.1 (2C), 130.7 (8C), 129.8 (4C), 128.2 (2C), 128.0 (4C), 126.4 (2C), 125.3 (2C), 123.9 (8C), 118.7 (2C), 111.8 (4C), 20.8 (4C). m/z calculated for $C_{53}H_{43}N_3O_2$ [M^+] exact mass= 753.3355, found MS (MALDI-TOF) = 753.3331.

Synthesis of SAM 3

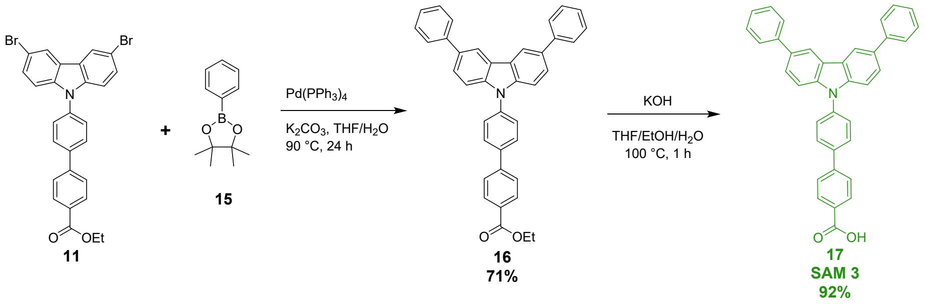

Scheme S3 Synthetic rout of SAM 3

Ethyl 4'-(3,6-diphenyl-9H-carbazol-9-yl)-[1,1'-biphenyl]-4-carboxylate (16)

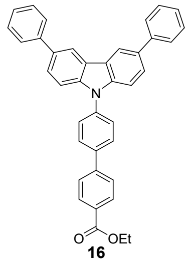

16

Procedure **2b** was carried out to give a 0.289 g of **16** (71%). **^{1}H NMR** (500 MHz, $CDCl_3$) $\delta_{(ppm)}$: 8.44 (2H, d, J = 1.8 Hz, 1 and 24-H_{Ar}), 8.20 (2H, d, J = 8.4 Hz, 12 and 13-H_{Ar}), 7.86 (2H, d, J = 8.5 Hz, 5 and 20-H_{Ar}), 7.79 -7.74 (6H, m, 8-10-H_{Ar} and 15-17-H_{Ar}), 7.70 (4H, td, J = 5.2, 2.6 Hz, 3, 6, 19 and 22-H_{Ar}), 7.56 – 7.49 (6H, m, 2, 7, 11, 14, 18 and 23-H_{Ar}), 7.38 (2H, t, J = 7.4 Hz, 4 and 21-H_{Ar}), 4.46 (2H, q, J = 7.1 Hz, -OCH_2), 1.47 (3H, t, J = 7.1 Hz, -CH_3). **^{13}C NMR** (126 MHz, $CDCl_3$) $\delta_{(ppm)}$: 166.6, 144.5, 141.9, 140.7 (2C), 139.2, 137.7, 134.0 (2C), 130.4 (2C), 129.8 (2C), 128.9 (2C), 128.9 (4C), 127.4 (4C), 127.3 (2C), 127.1 (2C), 126.8 (2C), 125.9 (2C), 124.3 (2C), 119.0 (2C), 110.3 (2C), 61.2, 14.3.

4'-(3,6-diphenyl-9H-carbazol-9-yl)-[1,1'-biphenyl]-4-carboxylic acid (SAM 3)

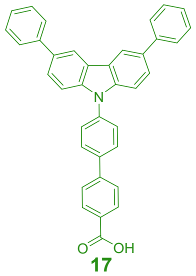

17
SAM 3

SAM 3 was obtained as a light-green solid (0.25 g, 92%), following the procedure **1d**. **SAM 3** was characterized by ^{1}H and ^{13}C NMR spectrometry as well by HRMS (Annex X) **^{1}H NMR** (400 MHz, DMSO) $\delta_{(ppm)}$: 8.76 (2H, d, *J* = 2.0 Hz, 1 and 24-H_{Ar}), 8.08 (4H, m, 5, 6, 19 and 21-H_{Ar}), 7.95 (2H, d, *J* = 8.6 Hz, 12 and 13-H_{Ar}), 7.86 – 7.78 (8H, m, 3, 8-10-H_{Ar} and 15-17, 22-H_{Ar}), 7.58 – 7.48 (6H, m, 2, 7, 11, 14, 18 and 23-H_{Ar}), 7.37 (2H, t, *J* = 7.4 Hz, 4 and 21-H_{Ar}). **^{13}C NMR** (101 MHz, DMSO) $\delta_{(ppm)}$: 167.1, 143.3, 140.7, 140.0 (2C), 138.4, 138.1 (2C), 132.7 (4C), 130.1, 129.9 (4C), 128.9 (6C), 128.8 (4), 126.9 (2C), 126.7 (2C), 125.5 (2C), 123.9 (2C), 119.1 (2C). m/z calculated for $C_{37}H_{25}NO_2$ [M^+] exact mass= 515.1885, found MS (MALDI-TOF) = 518.1886.

^{1}H and ^{13}C spectra

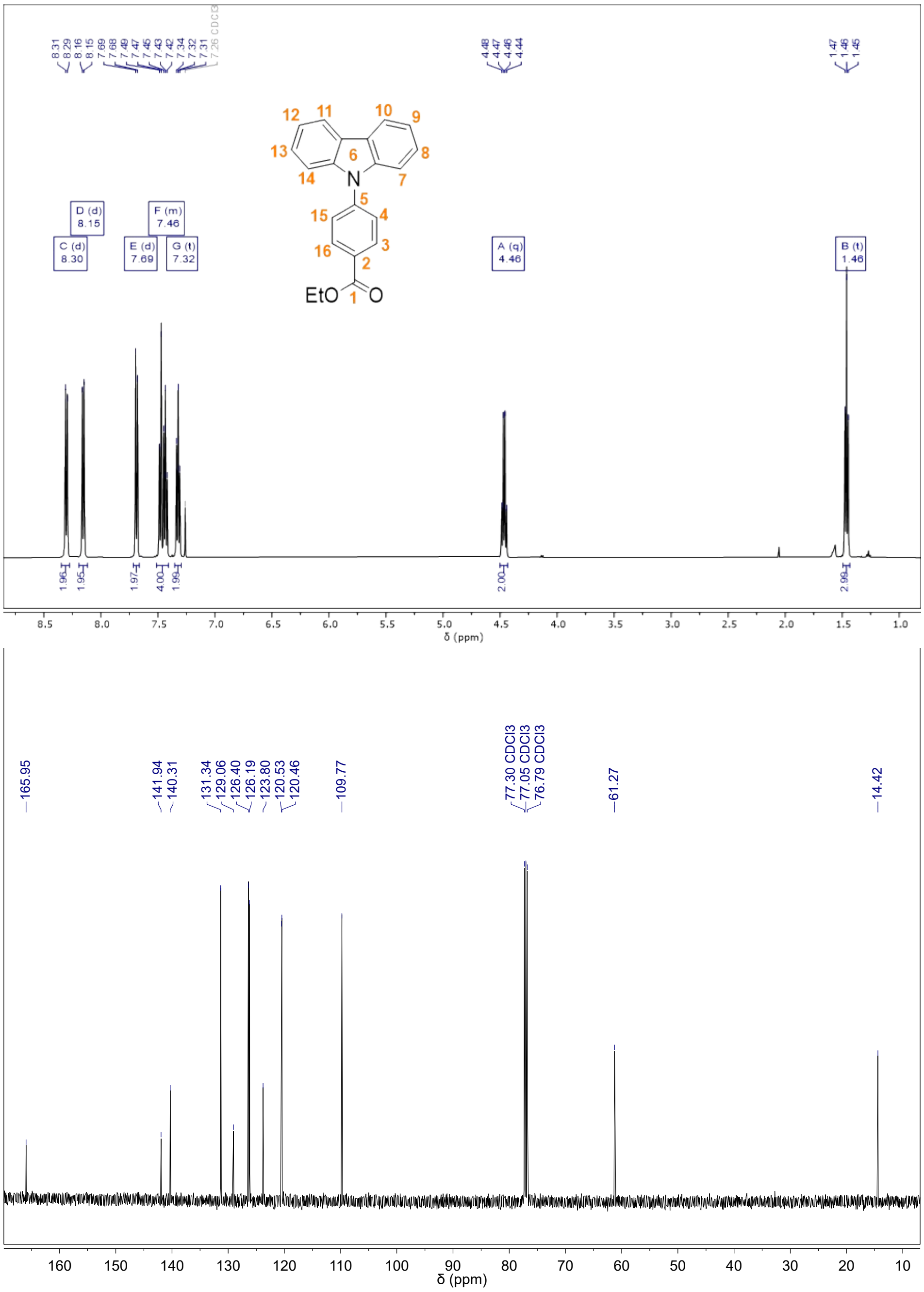

Figure S6. ^{1}H and ^{13}C NMRs of **3**

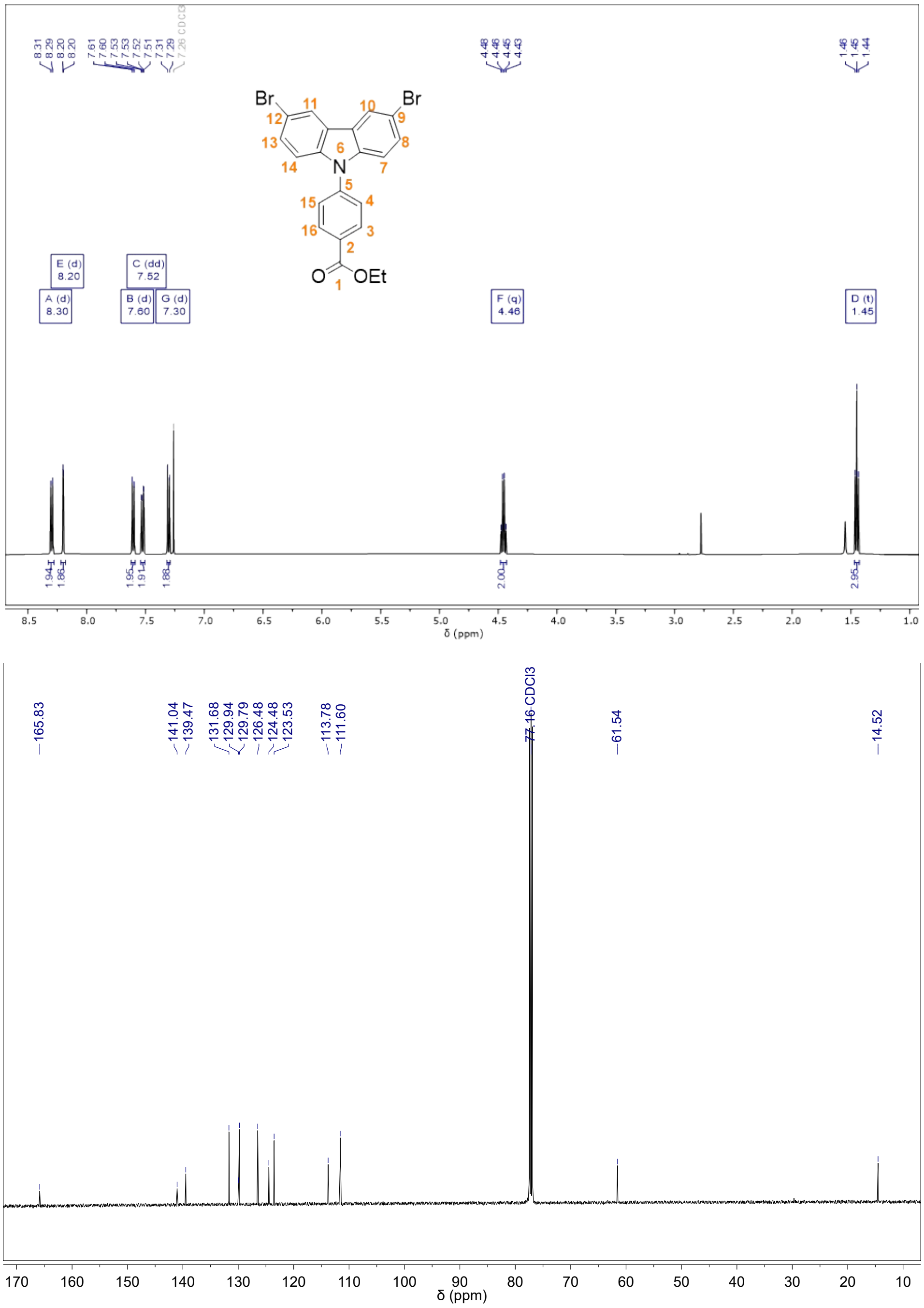

Figure S7. ^{1}H and ^{13}C NMRs of **4**

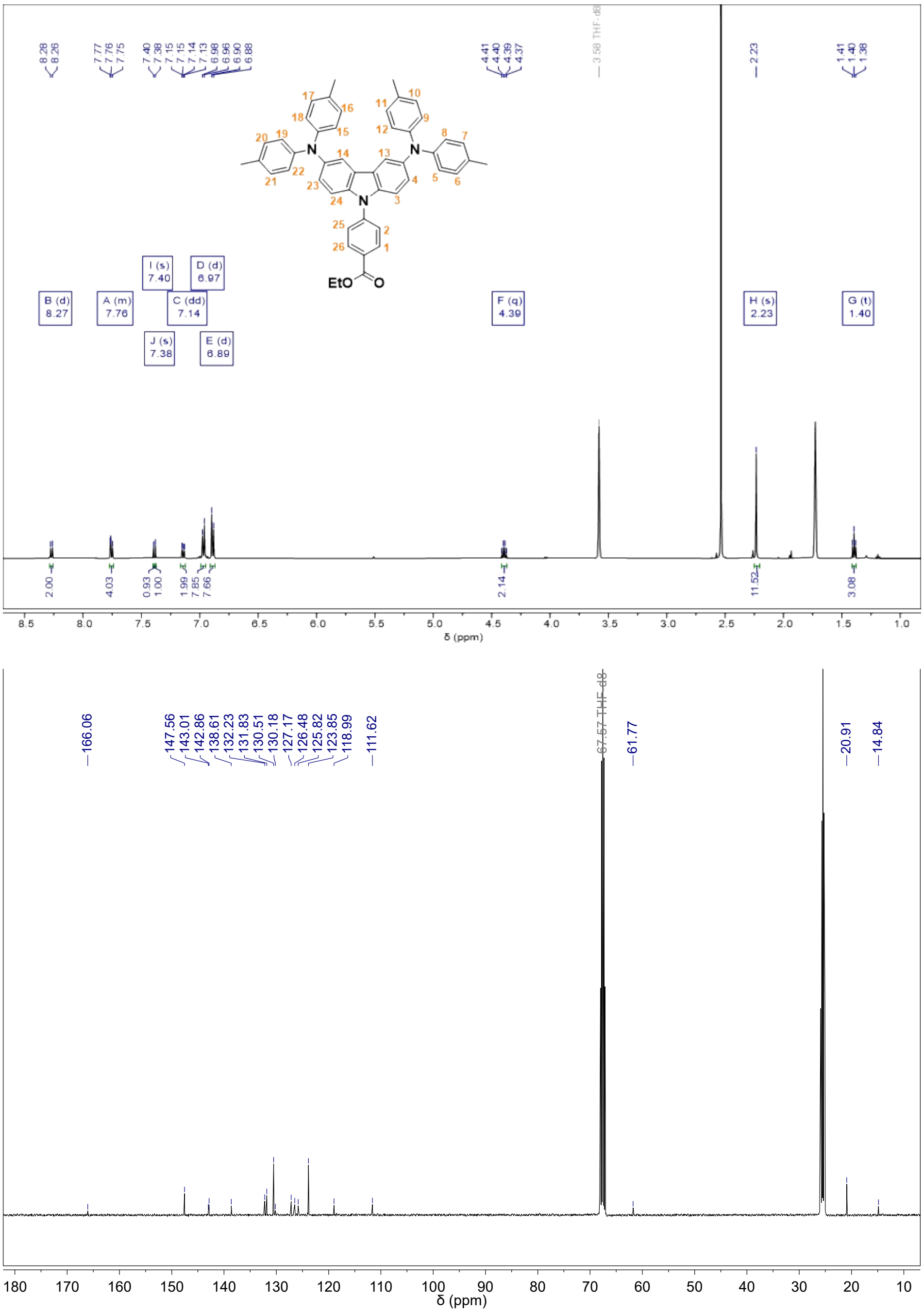

Figure S8. ^{1}H and ^{13}C NMRs of **6**

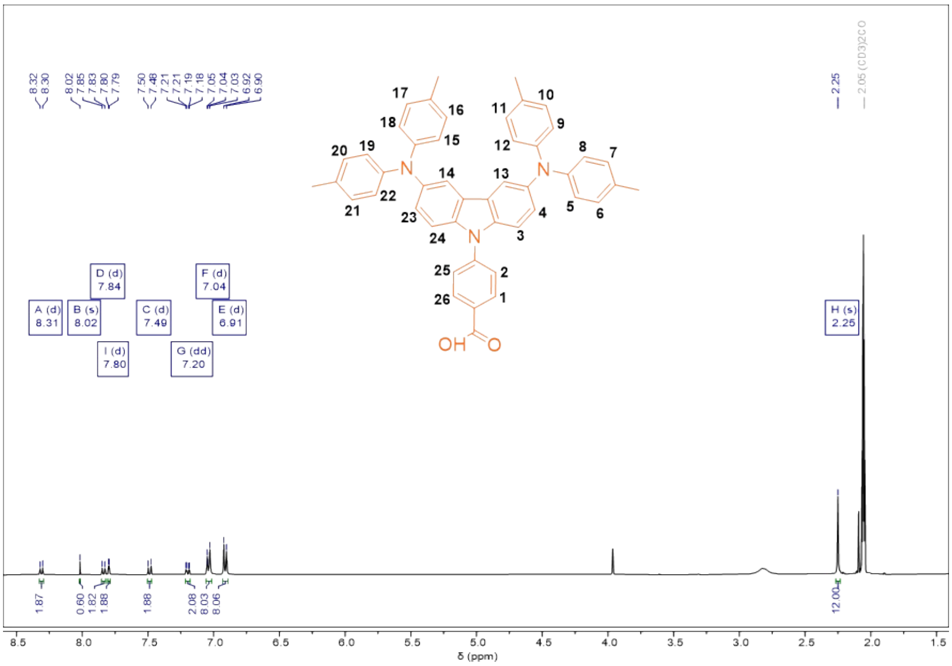

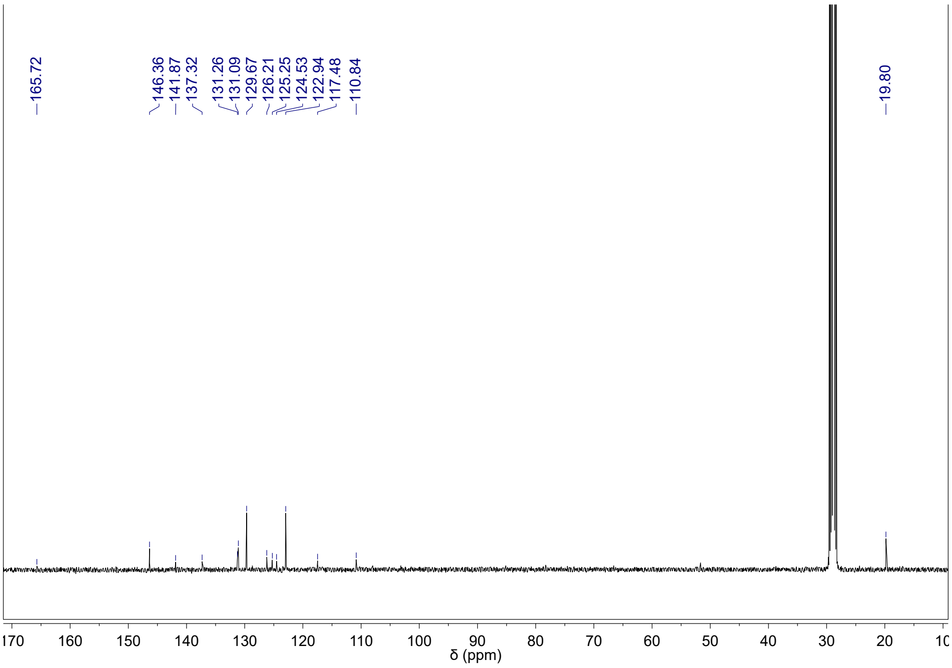

Figure S9. ^{1}H and ^{13}C NMRs of SAM 1 **(7)**

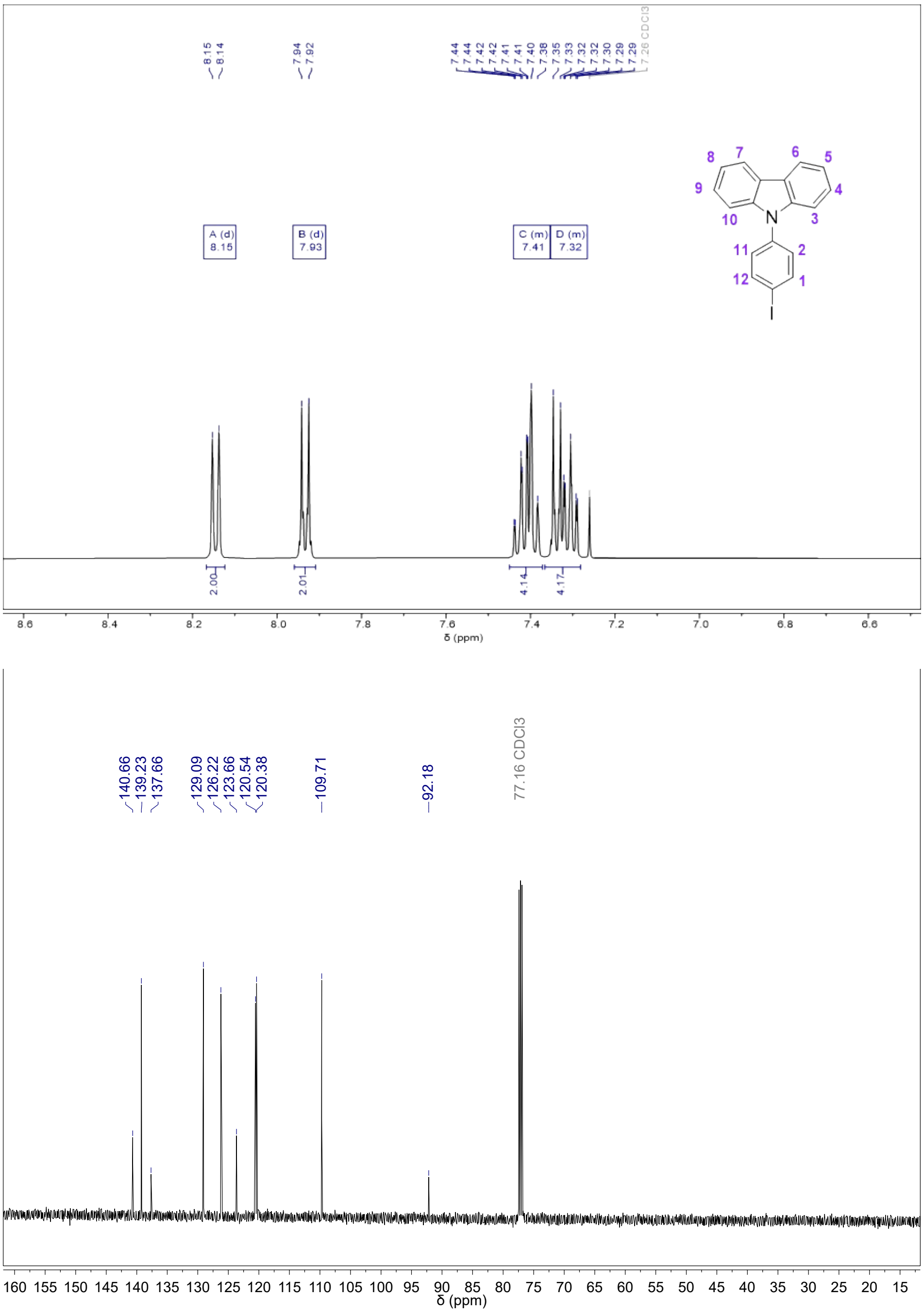

Figure S10. ^{1}H and ^{13}C NMRs of **9**

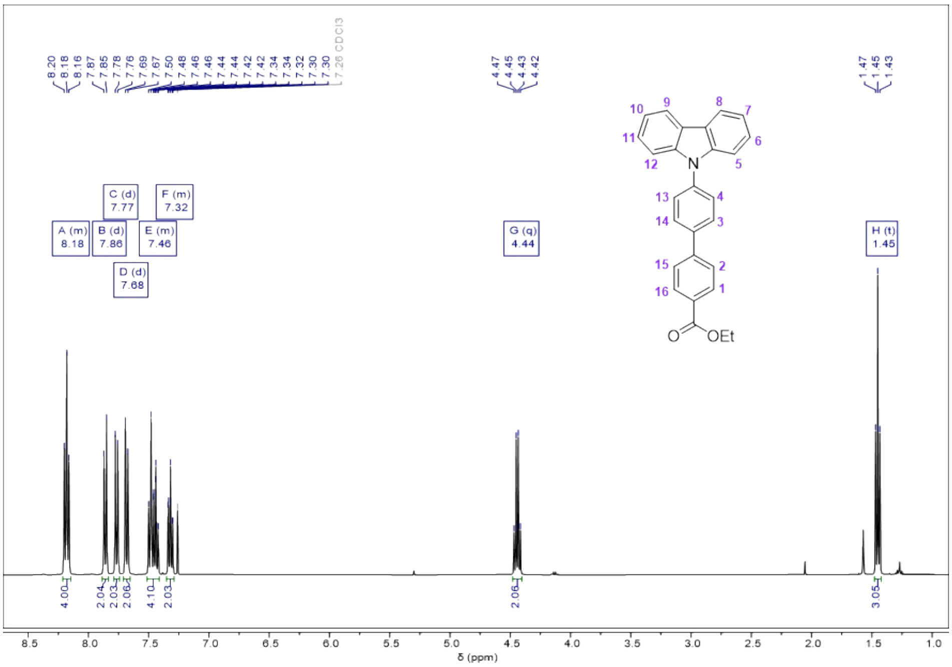

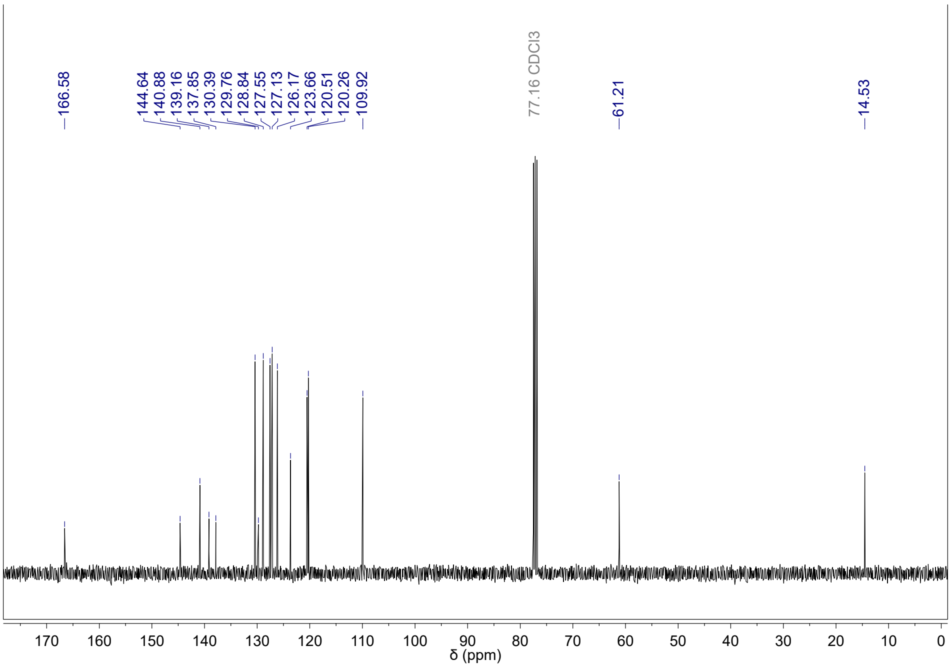

Figure S11. ^{1}H and ^{13}C NMRs of **11**

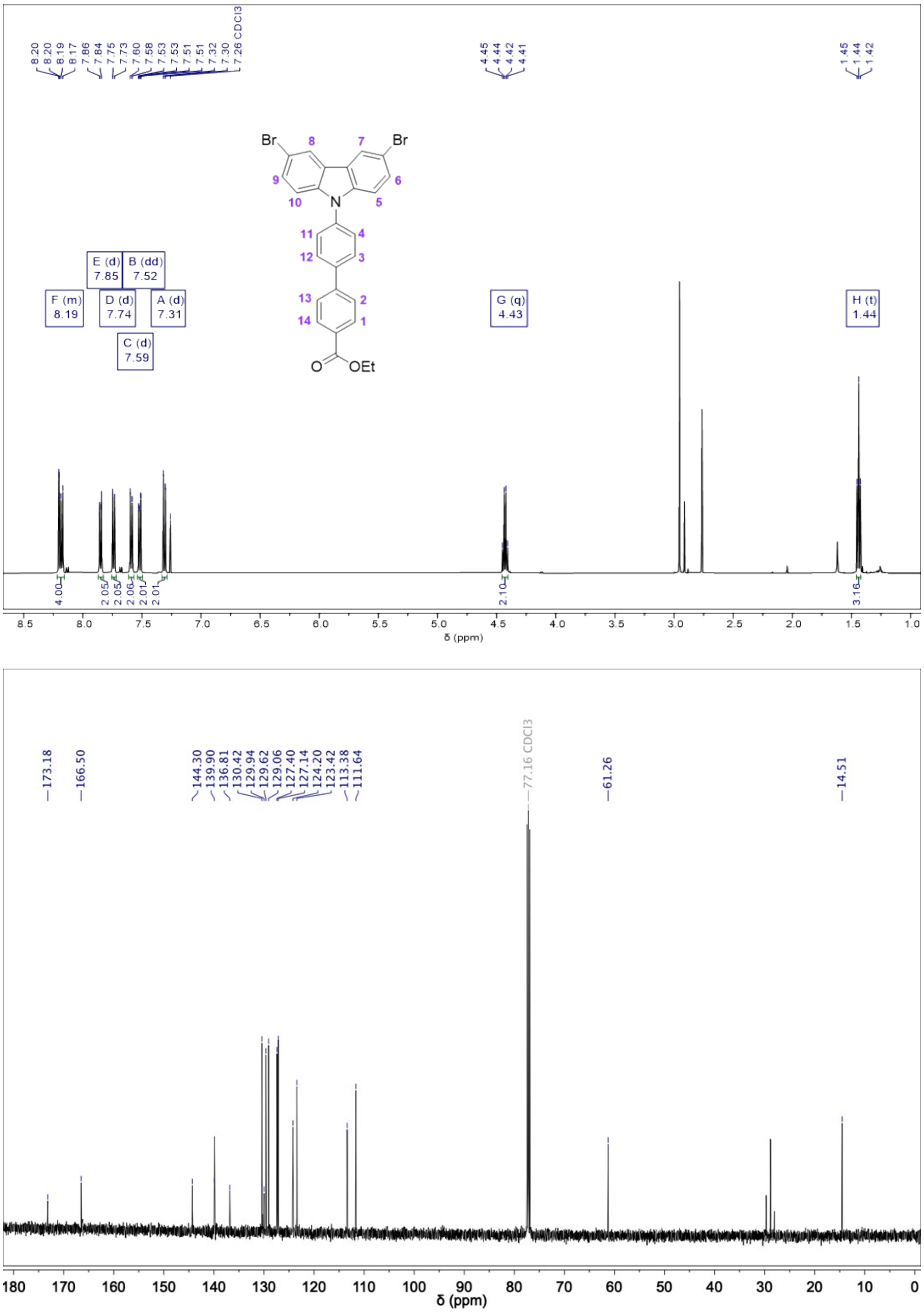

Figure S12. ^{1}H and ^{13}C NMRs of **12**

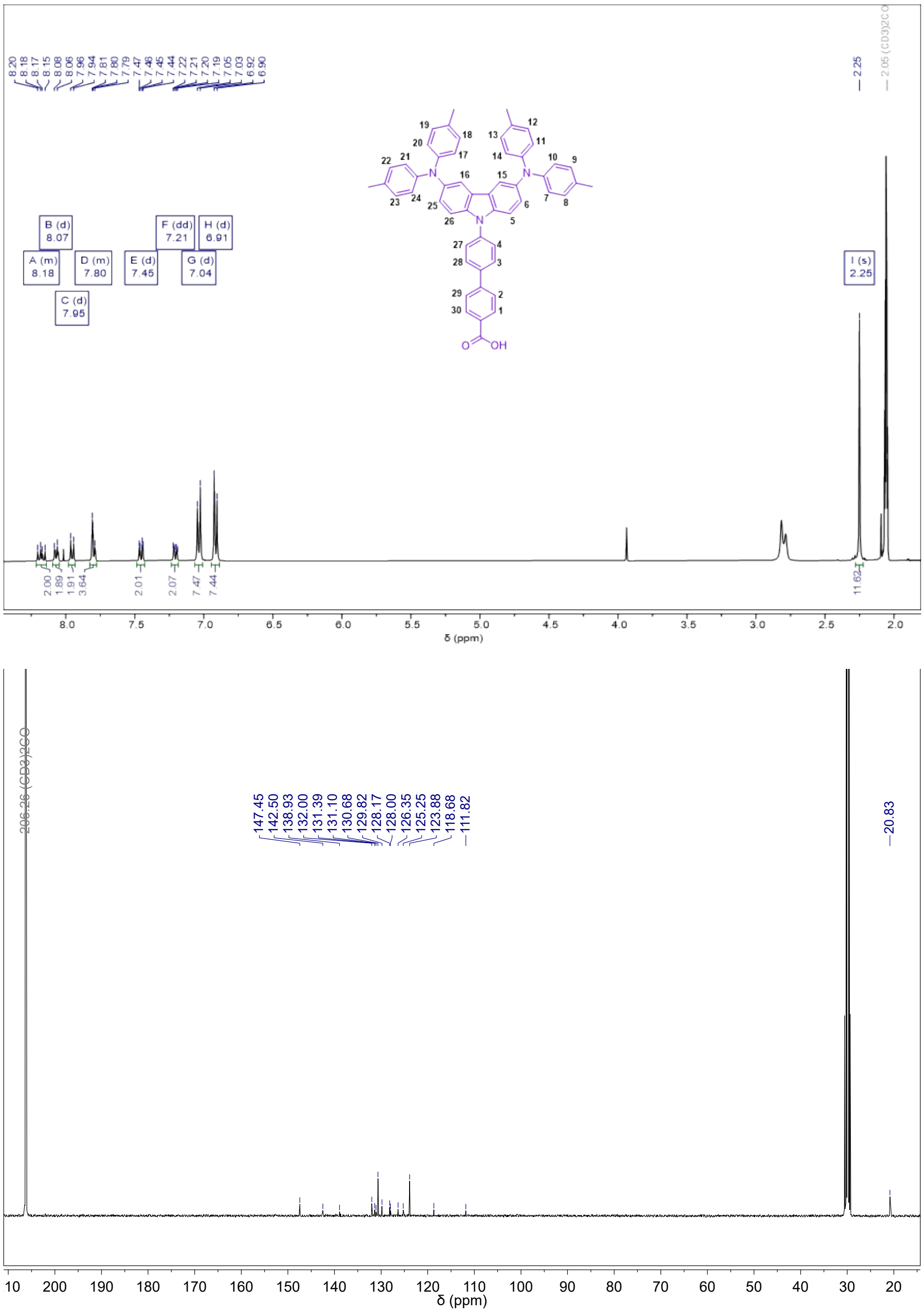

Figure S13. ^{1}H and ^{13}C NMRs of **14, SAM 2**

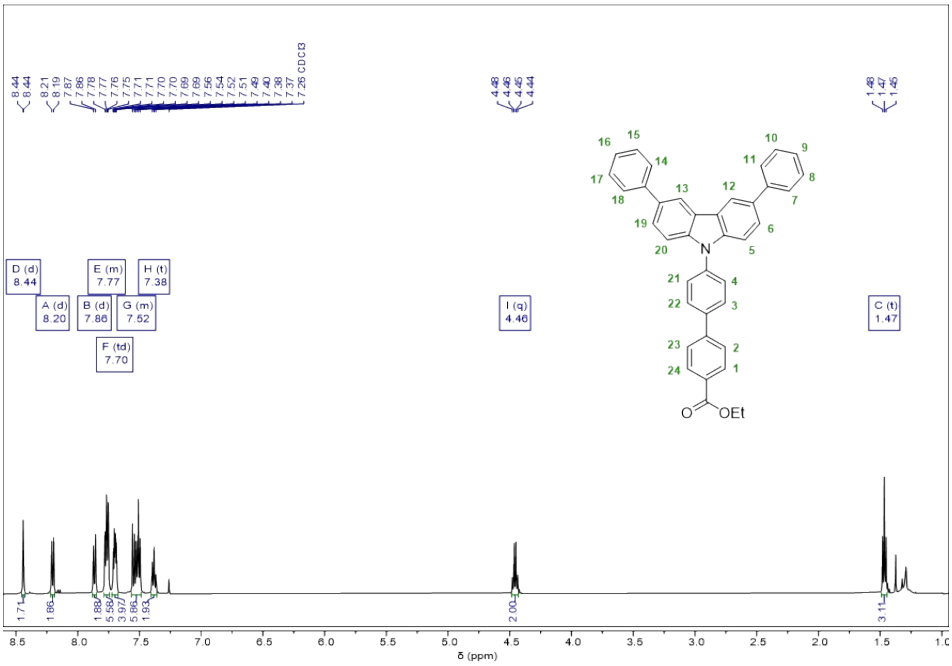

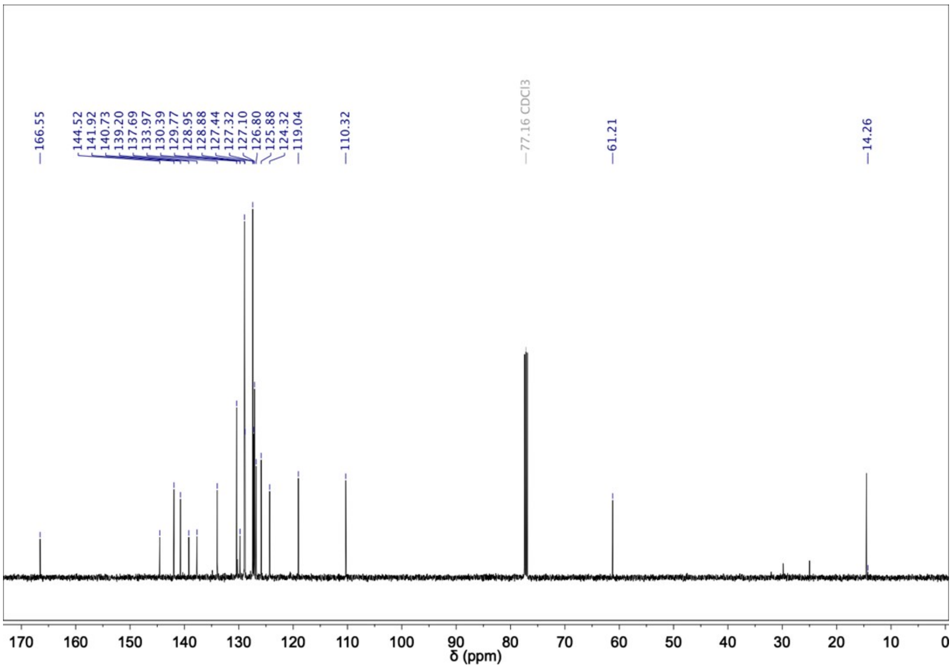

Figure S14. ^1H and ^{13}C NMRs of **16**

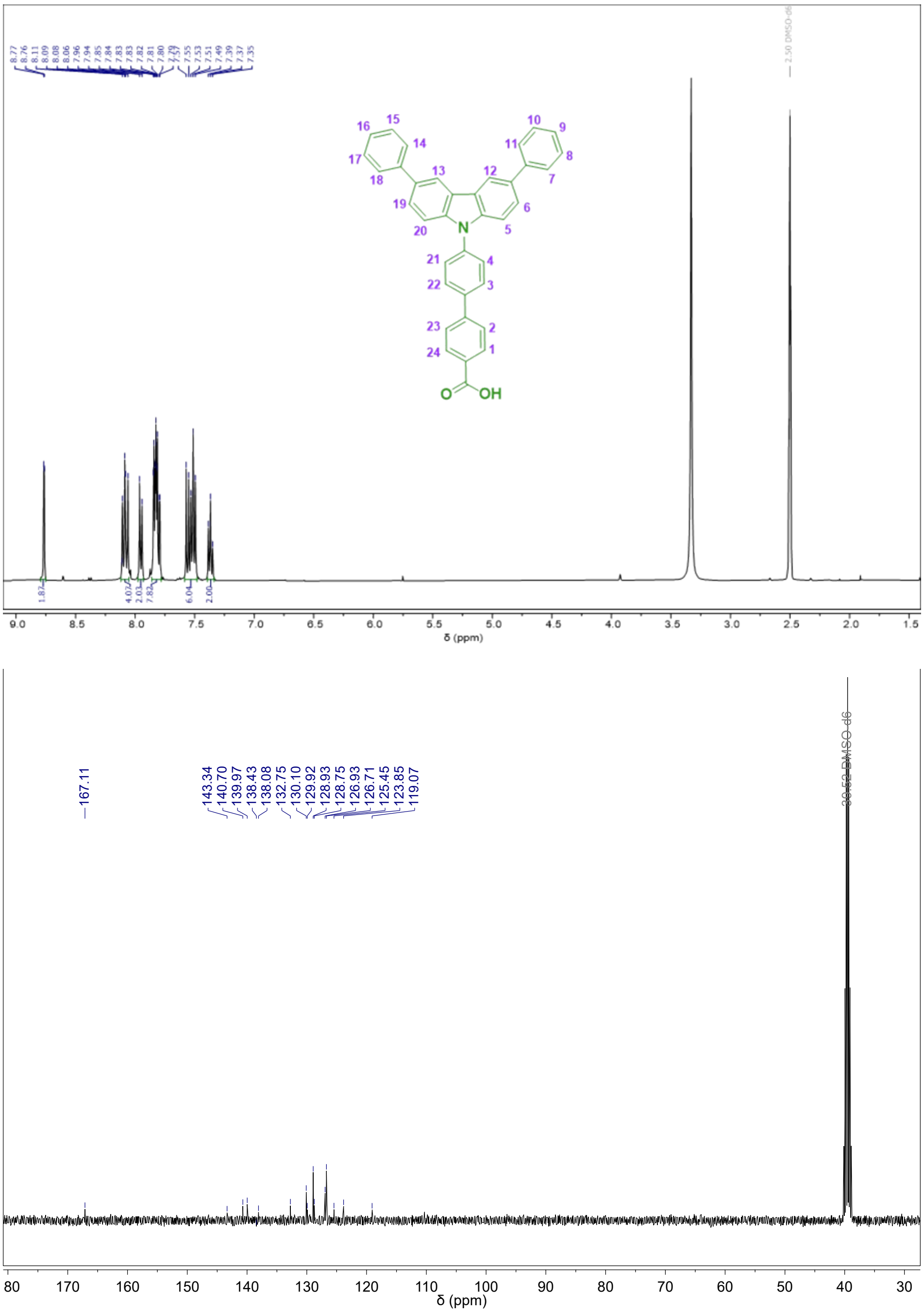

Figure S15. ^{1}H and ^{13}C NMRs of **17, SAM 3**

Mass Spectrum Spectra

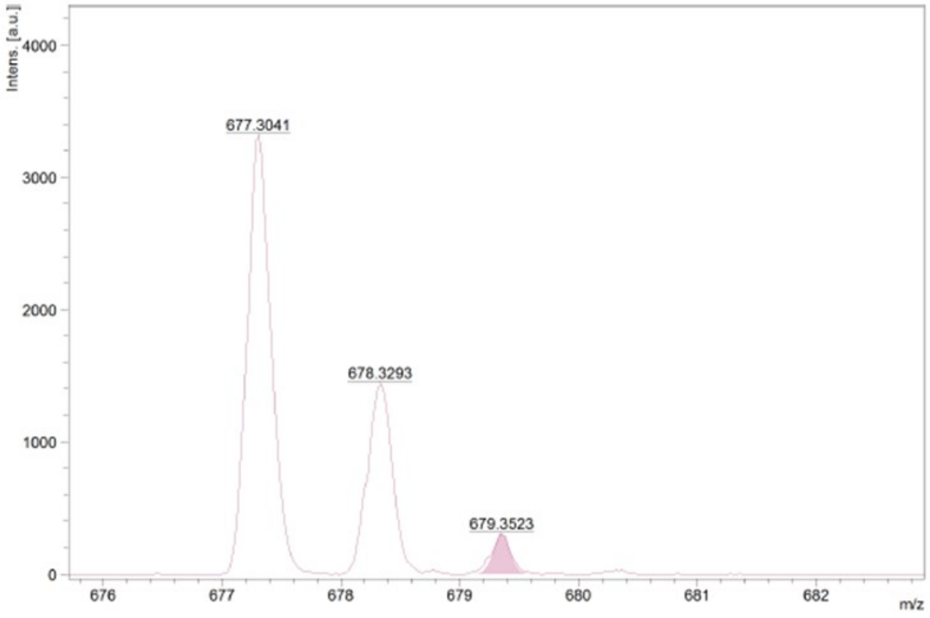

Figure S16. MS spectra of **SAM 1** (**7**)

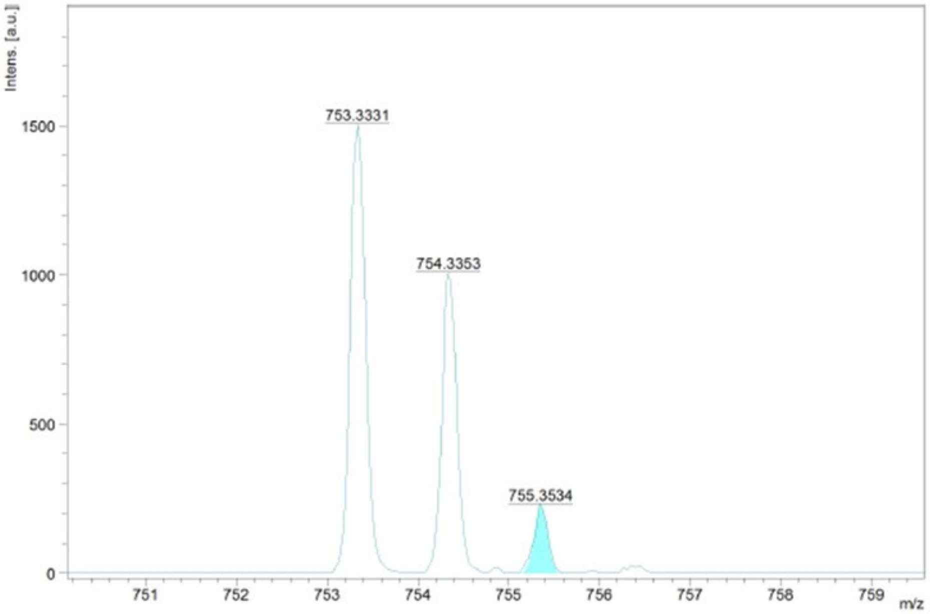

Figure S17. MS spectra of **SAM 2** (**14**)

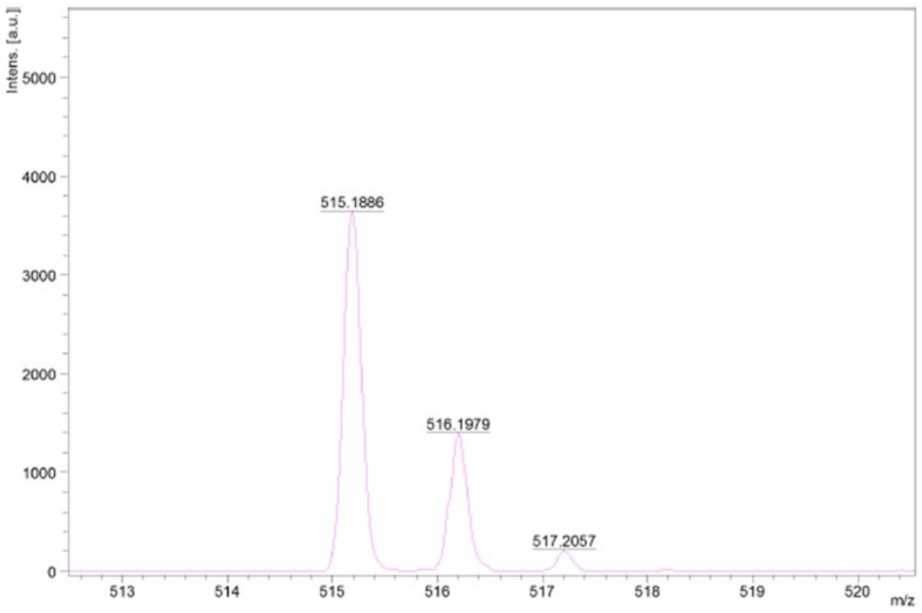

Figure S18. MS spectra of **SAM 3** (**17**)

REFERENCES

1. Li, W., Cariello, M., Méndez, M., Cooke, G. & Palomares, E. Self-Assembled Molecules for Hole-Selective Electrodes in Highly Stable and Efficient Inverted Perovskite Solar Cells with Ultralow Energy Loss. *ACS Appl Energy Mater* **6**, 1239–1247 (2023).

2. Saliba, M. *et al.* Cesium-containing triple cation perovskite solar cells: Improved stability, reproducibility and high efficiency. *Energy Environ Sci* **9**, 1989–1997 (2016).
